# Paraneoplastic Neurological Syndromes Associated with Ovarian Tumors: A Case Report and Systematic Review

**DOI:** 10.3390/diagnostics16162549

**Published:** 2026-08-12

**Authors:** Adrianna Zahorowska, Edyta Śliwak, Natalia Lerch, Karolina Engel-Eliasz, Adriana Szczęch, Mariola Krzyścin, Piotr Hajdasz, Aleksandra Marciniak, Agnieszka Brodowska, Andrzej Starczewski, Iwona Szydłowska

**Affiliations:** Department of Gynecology, Endocrinology and Gynecological Oncology, Pomeranian Medical University in Szczecin, 71-252 Szczecin, Polanddrkrzyscin@gmail.com (M.K.);

**Keywords:** paraneoplastic neurological syndromes, paraneoplastic cerebellar degeneration, onconeural antibodies, ovarian tumor

## Abstract

**Background:** Paraneoplastic neurological syndromes are rare immune-mediated disorders that may represent the first clinical manifestation of an underlying tumor. **Case Report:** This report describes a 58-year-old woman with rapidly progressive cerebellar dysfunction and anti-Yo antibodies in serum and cerebrospinal fluid. Definite PNS was diagnosed according to the PNS-Care Score criteria (score: 10/10). Oncological evaluation revealed FIGO IIIC high-grade serous ovarian carcinoma (HGSOC) with extensive peritoneal dissemination. Despite corticosteroids, cytoreductive surgery, and carboplatin–paclitaxel chemotherapy, neurological disability progressed, although tumor control was achieved. **Methods:** A PRISMA-guided review of PubMed and Scopus identified 33 studies of PNSs associated with ovarian tumors published between 2016 and 2026. **Results:** Anti-NMDAR encephalitis, mainly associated with ovarian teratomas, generally showed favorable recovery, whereas anti-Yo-associated cerebellar degeneration, predominantly linked to epithelial ovarian malignancies, had poor neurological outcomes. **Conclusions:** Prompt antibody testing, oncological investigation, and multidisciplinary treatment are essential, although anti-Yo-associated neurological deficits may be irreversible.

## 1. Introduction

Paraneoplastic neurological syndromes (PNSs) are rare immune-mediated disorders caused by an anti-tumor response directed against shared neuronal and tumor antigens. Unlike neurological complications related to direct tumor invasion, metastasis, metabolic disturbances, or treatment toxicity, PNSs represent remote effects of malignancy and may precede the diagnosis of the underlying neoplasm by weeks or months. Although uncommon, their clinical importance lies in the potential to serve as an early manifestation of occult cancer while simultaneously causing severe and often irreversible neurological injury [1].

Ovarian tumors are associated with distinct neurological paraneoplastic phenotypes that reflect complex interactions between tumor biology and host immunity. The spectrum is heterogeneous and spans both epithelial and germ-cell tumors, with clinical presentation largely determined by tumor histology and the associated antibody profile. High-grade serous ovarian carcinoma (HGSOC) is classically associated with paraneoplastic cerebellar degeneration (PCD), most often linked to anti-Yo antibodies, whereas ovarian teratomas are strongly associated with anti-N-methyl-D-aspartate receptor (anti-NMDAR) encephalitis. Less commonly, other neurological syndromes associated with antibodies such as anti-Ri, anti-CV2, anti-AMPAR, or seronegative immune-mediated syndromes have also been described [2,3].

Among these syndromes, PCD remains one of the most devastating neurological manifestations. It typically presents with subacute but rapidly progressive cerebellar dysfunction, including gait instability, limb and truncal ataxia, dysarthria, nystagmus, and diplopia. Although it occurs in fewer than 1% of patients with cancer, neurological recovery is often poor despite aggressive oncological treatment and immunotherapy. In contrast, anti-NMDAR encephalitis associated with ovarian teratomas frequently demonstrates substantial neurological improvement following early tumor resection and immunomodulatory therapy. This marked divergence in clinical course highlights the biological and prognostic heterogeneity of ovarian tumor-associated neurological PNSs [4,5].

Despite increasing awareness, these syndromes remain diagnostically challenging because neurological or psychiatric symptoms frequently precede tumor diagnosis and may initially mimic primary neurological disorders. Delayed recognition may postpone appropriate management, including oncological treatment, surgical intervention, and immunotherapy, thereby adversely affecting neurological outcomes. Furthermore, most available evidence is limited to isolated case reports and small case series, with relatively few attempts to systematically characterize the broader spectrum of ovarian tumor-associated neurological PNSs.

This report presents a case of anti-Yo-associated PCD as the initial manifestation of high-grade serous carcinoma. In addition, a systematic review of the literature was performed to characterize the clinical spectrum, antibody profiles, treatment strategies, and outcomes of PNSs associated with ovarian tumors.

## 2. Results

### 2.1. Case Description

**Figure 1:**
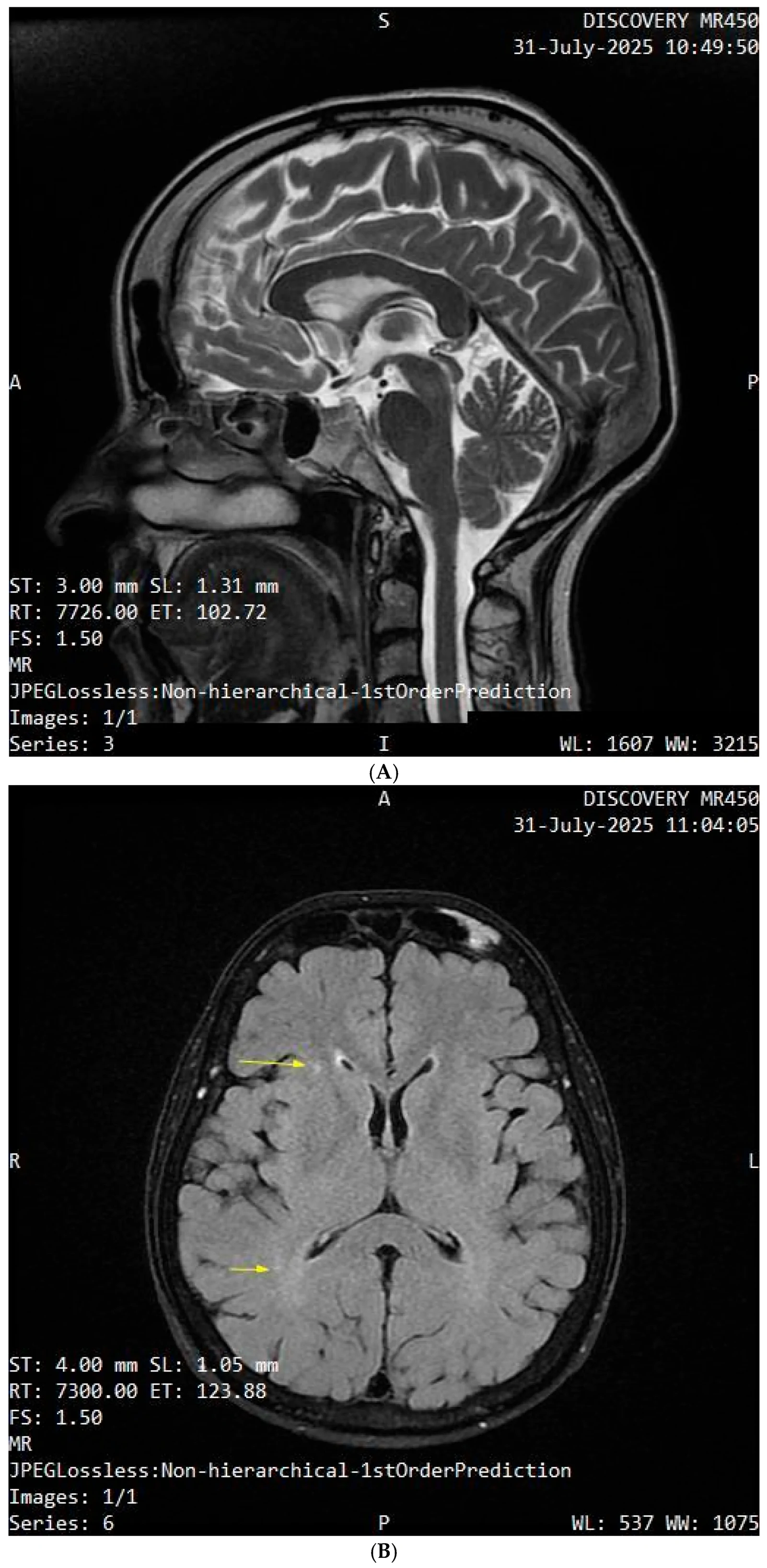
Initial brain magnetic resonance imaging (MRI, 1.5 T) performed on 31 July 2025. (**A**) Sagittal T2-weighted image demonstrating normal anatomical structures of the brainstem, fourth ventricle, and cerebellum without evidence of atrophy or focal lesions in the posterior fossa. (**B**) Axial T2-FLAIR image at the level of the lateral ventricles revealing isolated small hyperintense foci in the subcortical white matter (yellow arrows), consistent with mild chronic small-vessel ischemic changes (Fazekas grade 1), and moderate generalized cortical atrophy (GCA grade 2). No evidence of mass effect, diffusion restriction on DWI, microbleeds on SWI, or pathological brain contrast enhancement was observed.

**Figure 2:**
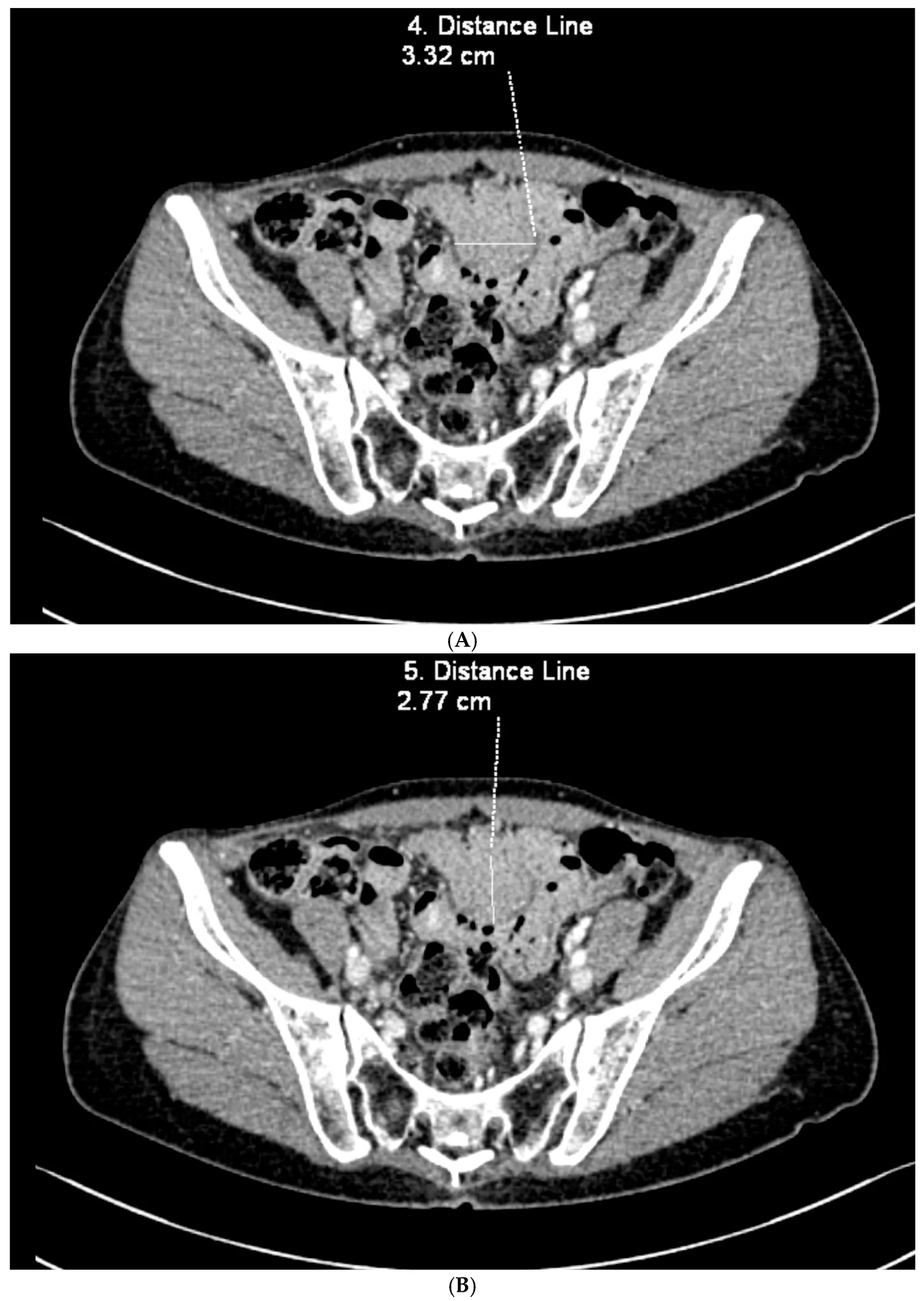
Contrast-enhanced computed tomography (CECT) of the abdomen and pelvis performed during the oncological work-up (11 September 2025). (**A**) Axial image (portal venous phase) demonstrating a pathologically enhancing tissue mass located superior to the uterine fundus, with a maximum transverse diameter of 3.32 cm (calliper). (**B**) Axial section showing the same adnexal lesion with a measured dimension of 2.77 cm (calliper).

A 58-year-old woman presented with rapidly progressive dysarthria, followed within several weeks by gait instability and impaired coordination. Acute cerebrovascular disease was excluded by head CT and CT angiography. During neurological hospitalization, she remained independently ambulatory but had an ataxic gait, scanning and intermittently explosive speech, four-limb dysmetria, a positive Romberg test, brisk deep tendon reflexes, and a tendency toward a left extensor plantar response. Based on the available documentation, her modified Rankin Scale score was retrospectively estimated as 2. As illustrated in Figure 1, brain MRI showed no structural cause of the cerebellar syndrome, revealing only mild small-vessel disease (Fazekas 1) and moderate generalized cortical atrophy. Cerebrospinal fluid analysis demonstrated lymphocytic pleocytosis of 31 cells/µL, a protein concentration of approximately 70 mg/dL, blood–cerebrospinal fluid barrier dysfunction, and oligoclonal bands. Anti-Yo1 antibodies were detected in both serum and cerebrospinal fluid. The high-risk cerebellar phenotype, high-risk antibody, and subsequently identified compatible malignancy fulfilled the criteria for definite paraneoplastic neurological syndrome, with a PNS-Care Score of 10/10. The patient received intravenous methylprednisolone without neurological improvement. Intravenous immunoglobulin and plasma exchange were not administered because, following the lack of response to corticosteroids, the treating team prioritized prompt tumor-directed treatment, specifically cytoreductive surgery, as the principal etiological treatment for the paraneoplastic syndrome. Initial oncological evaluation showed a CA-125 concentration of 37.50 U/mL and an HE4 concentration of 403 pmol/L. Pelvic ultrasonography suggested a 31 mm left ovarian lesion, whereas pelvic MRI demonstrated a 3.5 × 2.7 cm para-adnexal mass, confluent Douglas’ pouch deposits, interloop implants, and moderate ascites without a dominant ovarian tumor. As illustrated in Figure 2, abdominal CT confirmed these findings. During further diagnostic evaluation, CA-125 increased to 45.70 U/mL and HE4 to 544 pmol/L. Diagnostic laparoscopy with excision of a Douglas’ pouch lesion revealed HGSOC with CK20−, CK7+, ER+, PAX8+, WT1+, and CDX2− expression, and a Ki-67 proliferation index of approximately 50%. The patient underwent maximal-effort cytoreductive surgery, including hysterectomy with bilateral salpingo-oophorectomy, rectal resection, omentectomy, resection of bowel deposits, and pelvic lymph node excision. Complete macroscopic cytoreduction was not achieved because of extensive intraperitoneal dissemination, and residual macroscopic disease remained after surgery. Final histopathology established primary HGSOC pT3 N1a Mx, FIGO IIIC. Peritoneal and mesenteric deposits measured up to 3.5 cm and showed angioinvasion. One of four pelvic lymph nodes contained a 5 mm metastasis. Only a 2 mm focus of high-grade serous carcinoma was present in one ovary, while the contralateral ovary contained a 3 mm serous borderline tumor. Despite tumor-directed surgery, neurological disability continued to progress. At the first postoperative oncology assessment, CA-125 was 67.10 U/mL. Her World Health Organization/Eastern Cooperative Oncology Group performance status (WHO/ECOG PS) was formally assessed as 3–4, indicating severe functional impairment and confinement to bed or a chair for more than half of the day. Her modified Rankin Scale score was retrospectively estimated as 4. The patient received four cycles of carboplatin–paclitaxel chemotherapy. CA-125 subsequently decreased to 20.10 U/mL. A planned fifth cycle was not administered because of persistent WHO/ECOG PS 3–4, cachexia, muscle wasting, weight loss exceeding 10%, grade 2 neutropenia, and lack of neurological recovery. The risk of further treatment was considered to outweigh the potential benefit. At neurological follow-up, she was unable to walk or maintain an independent sitting position. Examination demonstrated head tremor, coarse multidirectional nystagmus with bilateral torsional components, severe four-limb ataxia, and scanning speech. Symptomatic treatment with gabapentin and baclofen partially reduced tremor but did not improve functional status. Follow-up CT showed no radiological evidence of active malignancy. Despite biochemical and radiological tumor control, neurological disability progressed to WHO/ECOG PS 4, with a retrospectively assessed modified Rankin Scale score of 5. At final follow-up, she remained bedridden, unable to communicate verbally, and under home hospice care. She was alive at manuscript submission, with persistent severe anti-Yo-associated paraneoplastic cerebellar degeneration. The clinical course, diagnostic work-up, oncological treatment, and neurological progression are summarized in Table 1.

**Figure 3:**
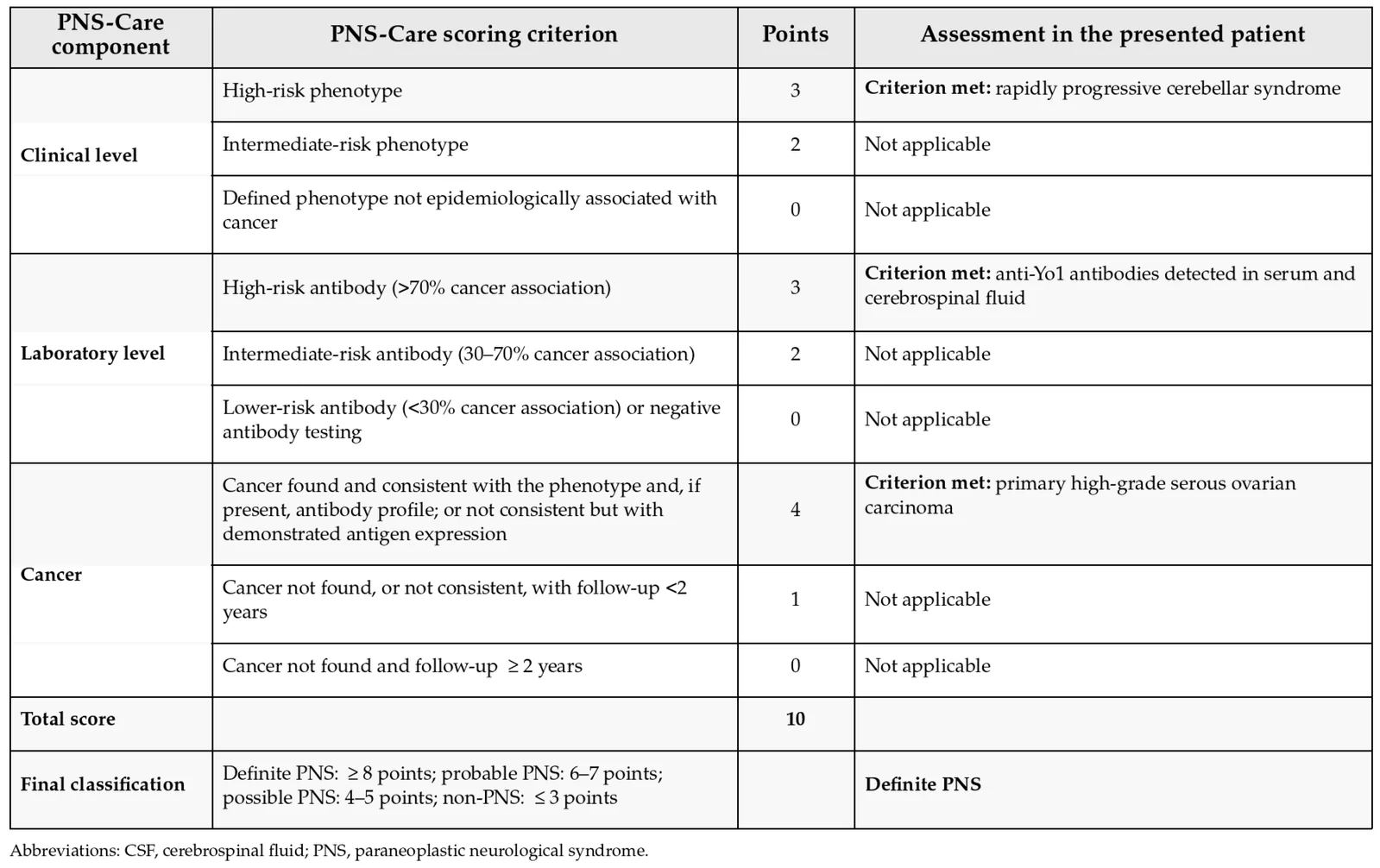
PNS–Care Score 2021 expanded to include the specific criteria met by the patient.

According to the updated 2021 PNS–Care Score criteria, illustrated in Figure 3, the patient was classified as having definite PNS based on the presence of a high-risk cerebellar phenotype, anti-Yo antibodies, and a compatible ovarian malignancy.

### 2.2. Systematic Review of the Literature

**Figure 4:**
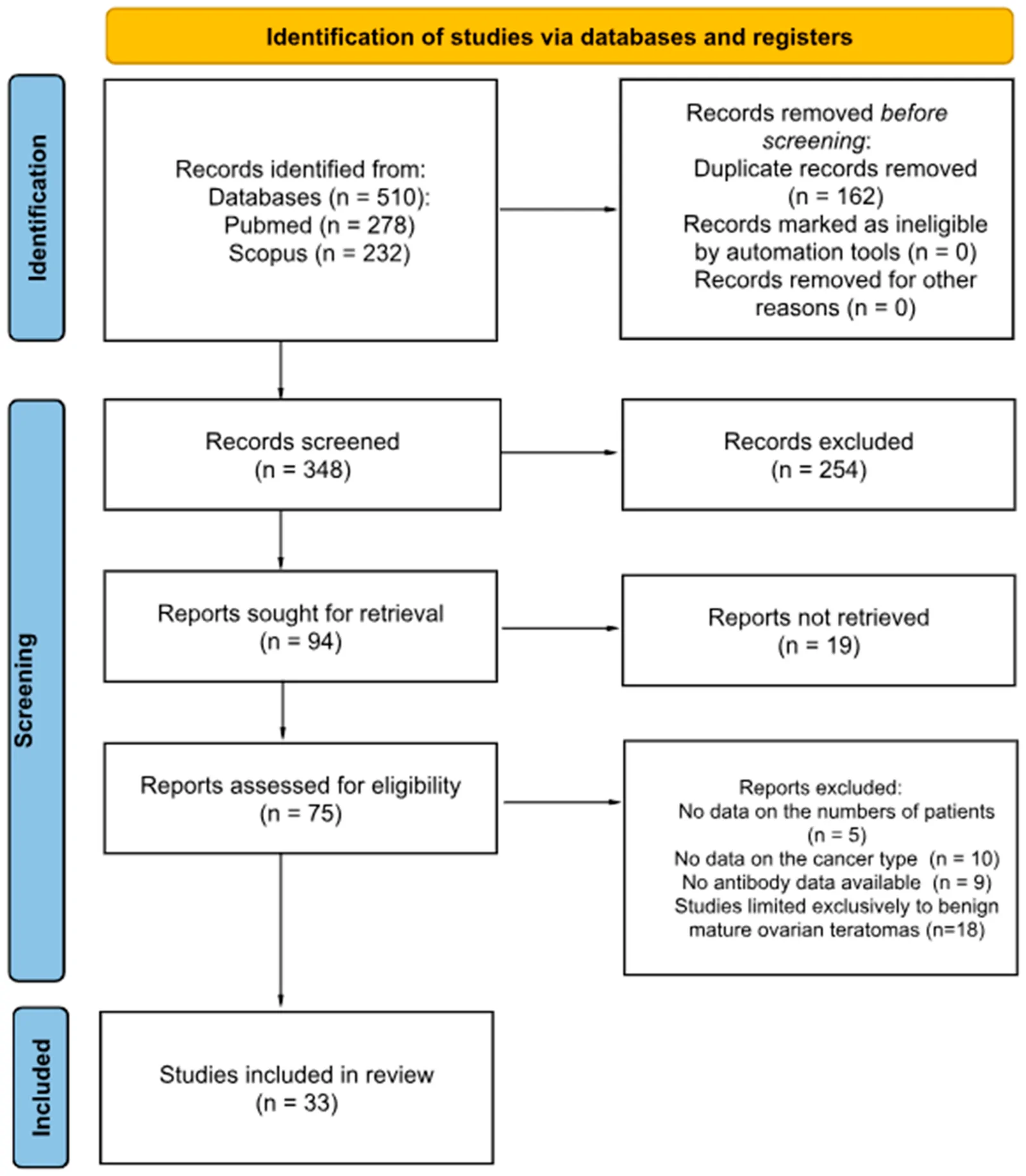
PRISMA flow diagram illustrating the study selection process for the systematic review of PNS associated with ovarian tumors.

The initial literature search identified 510 records. After removing 162 duplicates, 348 records were screened based on titles and abstracts. Full–text assessment was performed for 75 potentially eligible studies, of which 42 were excluded with specific reasons. Ultimately, 33 studies met the inclusion criteria and were included in the final qualitative synthesis. The study selection process is presented in the PRISMA flow diagram (Figure 4). The included studies consisted predominantly of case reports, case series, and observational cohort studies describing immune–mediated neurological syndromes associated with ovarian neoplasms. Substantial heterogeneity was observed in both clinical presentation and immunological profiles. The findings were organized according to antibody profile, tumor histology, and neurological phenotype. The most frequently reported syndromes included anti-NMDAR encephalitis and PCD and, less commonly, opsoclonus–myoclonus syndrome, sensory neuropathies, trigeminal neuropathy, and other antibody-associated autoimmune neurological syndromes.

Anti–NMDAR encephalitis and ovarian germ–cell tumors. Anti–NMDAR encephalitis was predominantly associated with ovarian teratomas, particularly mature and immature teratomas, and typically presented with psychiatric manifestations, seizures, encephalopathy, movement disorders, memory impairment, and altered consciousness. These patients generally demonstrated favorable neurological outcomes following tumor resection combined with immunotherapy.

Anti–Yo–associated PCD and epithelial ovarian malignancies. Anti–Yo–associated syndromes were primarily observed in patients with epithelial ovarian malignancies, particularly high-grade serous ovarian carcinoma, and most commonly manifested as progressive cerebellar dysfunction characterized by ataxia, dysarthria, nystagmus, diplopia, and gait instability. Neurological recovery in this group was generally poor despite aggressive oncological treatment and immunomodulatory therapy.

Other antibody profiles and neurological syndromes. Less frequently reported antibody profiles included anti-Ri, anti-Hu, anti-CV2, anti-Ro52, anti-AMPAR, anti-Tr/DNER, and anti-recoverin antibodies, reflecting the broader immunological spectrum of ovarian tumor-associated neurological syndromes. Treatment strategies across studies commonly included tumor–directed therapy (surgery and/or chemotherapy) combined with immunotherapy, including corticosteroids, intravenous immunoglobulin (IVIG), plasmapheresis, rituximab, and cyclophosphamide.

Overall, neurological outcomes varied considerably depending on the underlying syndrome and antibody profile. Anti-NMDAR-associated cases were associated with the most favorable prognosis, whereas anti-Yo–associated PCD demonstrated persistently poor neurological outcomes, frequently resulting in severe disability or death despite multimodal treatment. Because of substantial heterogeneity in study design, sample size, follow–up duration, and outcome reporting, pooled descriptive statistics were not considered sufficiently reliable and were therefore not presented. The detailed characteristics of included studies are summarized in Table 2 and Table 3. Results of the structured JBI critical appraisal are presented in Supplementary Table S2.

## 3. Discussion

### 3.1. Clinical and Immunopathogenic Spectrum

Paraneoplastic neurological syndromes (PNSs) are rare immune–mediated complications of malignancy that may precede tumor diagnosis and substantially complicate the diagnostic process [1,38]. The findings of the present review indicate that ovarian tumor–associated neurological syndromes constitute a clinically and biologically heterogeneous group differing in tumor association, antibody profile, treatment response, and neurological outcome.

Two predominant clinicopathological patterns emerged from the present review. Ovarian tumor-associated anti-NMDAR encephalitis occurred mainly in younger women with ovarian teratomas, whereas anti-Yo–associated paraneoplastic cerebellar degeneration (PCD) was predominantly linked to epithelial ovarian malignancies, particularly high–grade serous ovarian carcinoma [37,39,40,41]. Previous systematic reviews support this distinction: approximately 71% of reported ovarian cancer-associated PCD cases were associated with high–grade serous carcinoma, whereas patients with teratoma-associated anti-NMDAR encephalitis had a mean age of approximately 24 years, with mature teratomas representing the predominant histological subtype [40,41].

**Figure 5:**
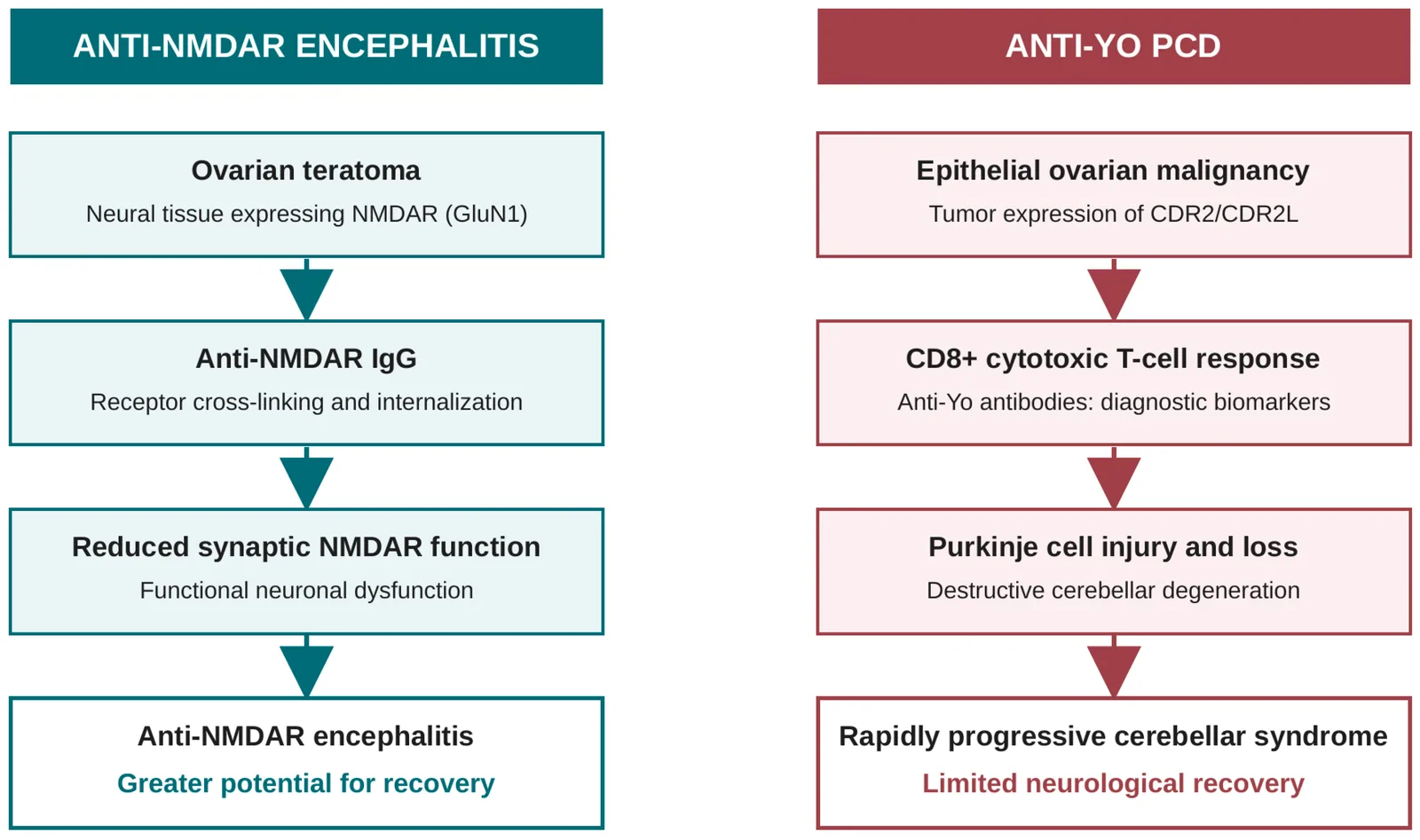
Comparison of the immunopathogenic mechanisms underlying ovarian teratoma-associated anti-NMDAR encephalitis and anti-Yo–associated paraneoplastic cerebellar degeneration.

These clinical differences likely reflect distinct immunopathogenic mechanisms. In ovarian teratoma–associated anti-NMDAR encephalitis, ectopic neural tissue expressing NMDA receptor antigens may initiate an antibody-mediated immune response that alters neuronal receptor function [22,42]. By contrast, anti-Yo–associated PCD is primarily associated with cytotoxic T–cell–mediated injury directed against Purkinje cells and is therefore more likely to cause irreversible neuronal loss [43,44]. Although CDR2 was traditionally considered the principal anti-Yo antigen, CDR2L may represent a more relevant target because it is expressed in both tumor tissue and Purkinje cells [44]. These contrasting immunopathogenic pathways are summarized in Figure 5.

The underlying mechanisms are reflected in treatment response and prognosis. Patients with ovarian teratoma-associated anti-NMDAR encephalitis frequently experience substantial neurological recovery following tumor removal and immunotherapy [24,25,32,33,39]. Conversely, anti-Yo–associated PCD usually presents with rapidly progressive gait ataxia, dysarthria, and limb incoordination and is frequently associated with severe persistent disability despite immunotherapy and tumor–directed treatment [6,11,20,43]. The course of the present patient, who had HGSOC, was consistent with this pattern: biochemical and radiological tumor control was achieved, but neurological disability continued to progress. Although isolated cases of neurological improvement after early combined oncological and immunomodulatory treatment have been reported, such recovery appears uncommon [9].

Less frequent phenotypes and antibody associations identified in the review included anti-Ri, anti-Hu, anti-CV2/CRMP5, anti-AMPAR, anti-Tr/DNER, and anti-recoverin–associated syndromes [10,14,15,18,45]. This broader spectrum supports phenotype–directed diagnostic evaluation, rather than testing limited exclusively to anti-Yo and anti-NMDAR antibodies.

### 3.2. Clinical Implications

Neuronal antibody testing should be considered in patients with a subacute or rapidly progressive cerebellar syndrome that remains unexplained after exclusion of common structural, vascular, infectious, metabolic, and toxic causes. Testing should also be considered in patients with suspected autoimmune encephalitis presenting with psychiatric symptoms, seizures, movement disorders, altered consciousness, or autonomic instability [46].

Antibody selection should be guided by the neurological phenotype. In rapidly progressive cerebellar syndromes, testing may include anti-Yo, anti-Hu, anti-Ri, anti-CV2/CRMP5, anti-Ma2, amphiphysin, and anti-Tr/DNER antibodies. In encephalitic, psychiatric, or seizure–related presentations, neuronal surface antibodies, particularly anti-NMDAR and anti-AMPAR, should also be assessed. Whenever possible, testing should be performed in paired serum and cerebrospinal fluid samples. Unexpected results, particularly isolated or low-titer findings obtained using commercial panels, should be confirmed using an alternative method and interpreted in the context of the clinical phenotype [46].

Oncological investigation should be initiated promptly and directed by the phenotype and antibody–cancer association. Women with anti-Yo–associated PCD require gynecological and breast assessment, whereas pelvic imaging should be obtained early in suspected ovarian teratoma-associated anti-NMDAR encephalitis. When initial cancer screening is negative in a patient with both a high-risk phenotype and a high-risk antibody, screening should be repeated every 4–6 months for 2 years, with the interval individualized according to clinical evolution and patient-specific risk factors [47].

In the present patient, the combination of a high-risk neurological phenotype, a high-risk anti-Yo antibody, and a compatible malignancy resulted in a PNS–Care Score of 10/10, meeting the criteria for definite PNS [47,48,49]. Management should involve early collaboration among neurologists or neuroimmunologists, gynecologic and medical oncologists, radiologists, pathologists, and laboratory immunologists. Rehabilitation and palliative care specialists should also be involved according to the severity and progression of neurological disability.

### 3.3. Future Directions

Future research should focus on biomarkers that enable earlier recognition of neuronal injury and the underlying malignancy. Potential candidates include longitudinal serum–cerebrospinal fluid antibody profiles, markers of neuroaxonal injury, CDR2/CDR2L expression patterns, and circulating tumor-derived biomarkers [1,5,38]. Prospective multicenter studies and standardized biobanks are required to determine whether these markers can predict neurological progression, treatment response, or the presence of an occult tumor before it becomes detectable using conventional imaging.

Novel immunotherapeutic strategies should account for the different mechanisms underlying these syndromes. In refractory anti-NMDAR encephalitis, further investigation of B–cell-, plasma–cell-, and cytokine–directed treatments is warranted [1]. For anti-Yo–associated PCD, research should focus on strategies capable of controlling cytotoxic T–cell responses before irreversible Purkinje cell loss occurs [38,43,44]. Because current evidence is derived mainly from small observational studies, registries and multicenter prospective trials are required before such approaches can be incorporated into routine clinical practice.

### 3.4. Limitations

Several limitations should be acknowledged. Most included publications were case reports, case series, or small observational studies, resulting in substantial heterogeneity in diagnostic methods, treatment strategies, follow-up duration, and outcome reporting. Publication bias is likely because severe, atypical, or clinically successful cases may be more frequently reported. Seronegative and unrecognized cases may also contribute to underestimation of the true incidence of these syndromes.

The search was restricted to PubMed and Scopus and to publications from January 2016 to 23 March 2026. Consequently, relevant historical reports and studies indexed in other databases may have been missed. Although this period was selected to reflect contemporary antibody testing, tumor classification, imaging, and management, the restriction is particularly important in the context of rare disorders. Earlier foundational publications and more recent sources cited to contextualize the findings were not treated as studies included in the systematic synthesis. The review was not prospectively registered in PROSPERO. Finally, the heterogeneity and predominantly descriptive nature of the available evidence precluded a meaningful quantitative synthesis.

Despite these limitations, these findings emphasize the close relationship between neurological phenotype, antibody profile, and tumor histology. Early phenotype-directed antibody testing, prompt oncological investigation, and multidisciplinary management remain essential, although neurological recovery in anti-Yo–associated PCD is often limited even when tumor control is achieved.

## 4. Materials and Methods

### 4.1. Case Description

The presented case involved a 58-year-old woman with anti-Yo–associated PCD who was referred to the University Clinical Hospital No. 1 of the Pomeranian Medical University in Szczecin, Poland, for evaluation of progressive neurological symptoms. Neuroimaging, including computed tomography (CT) and brain MRI, excluded acute intracranial pathology; brain MRI demonstrated only nonspecific chronic vascular changes. Cerebrospinal fluid analysis demonstrated lymphocytic pleocytosis, elevated protein concentration, and the presence of anti-Yo antibodies. Subsequent histopathological examination confirmed HGSOC. The study was conducted in accordance with the principles of the Declaration of Helsinki. Written informed consent was obtained from the patient for publication of this case report and accompanying clinical data.

### 4.2. Systematic Review

A systematic literature review was conducted in accordance with the Preferred Reporting Items for Systematic Reviews and Meta-Analyses (PRISMA) guidelines. To maximize search sensitivity and minimize the risk of missing relevant studies, a broad search strategy was applied. The literature search was conducted on 23 March 2026, and included studies published from 1 January 2016 to 23 March 2026 using the MEDLINE (via PubMed) and Scopus databases. The literature search was restricted to publications from 2016 to 2026 to provide a synthesis of contemporary evidence and to reduce heterogeneity related to historical differences in neuronal antibody testing, neuroimaging, histopathological classification, diagnostic criteria, and oncological management. The selected period also encompasses the introduction of the updated 2021 diagnostic criteria for paraneoplastic neurological syndromes, including the PNS–Care Score, which constitutes the current diagnostic framework used to interpret the included cases. The search strategy combined free-text keywords and Boolean operators related to PNS, autoimmune neurological disorders, and gynecologic malignancies, using the following Boolean query: (“paraneoplastic syndrome*” OR “paraneoplastic neurologic syndrome*” OR “anti-NMDAR encephalitis” OR “cerebellar degeneration” OR “autoimmune disease*” OR “autoimmune response*” OR “tumor-associated antibod*” OR “onconeural antigen*”) AND (“gynecologic neoplasm*” OR “ovarian neoplasm*” OR “ovarian carcinoma” OR “ovarian cancer” OR “primary peritoneal carcinoma” OR “peritoneal carcinoma”). The complete database-specific search strategies, including the exact queries used for PubMed and Scopus and the number of records retrieved from each database, are presented in Supplementary Table S1. Two independent reviewers screened titles, abstracts, and full-text articles for eligibility and independently extracted relevant study data. Any disagreements regarding study selection or data extraction were resolved through discussion until consensus was reached. The study selection process is presented in Figure 4.

A total of 510 records were identified. After duplicate removal and initial screening, the titles and abstracts were assessed for relevance based on predefined inclusion and exclusion criteria. Eligible studies included original clinical studies, observational studies, case series, and case reports describing immune-mediated neurological syndromes associated with ovarian neoplasms, including epithelial ovarian malignancies and ovarian teratomas. Studies reporting exclusively benign mature ovarian teratomas were excluded, whereas studies including immature teratomas remained eligible, even when mature and immature teratomas were analyzed within the same cohort. Studies were excluded if they focused exclusively on non–neurological paraneoplastic manifestations; lacked extractable clinical data; were review articles, conference abstracts, editorials, or non–English publications; or represented duplicate reports. Following full–text assessment, 33 studies met the inclusion criteria and were included in the final qualitative synthesis. Given the heterogeneity of the included studies, which consisted predominantly of case reports and observational data, a formal meta–analysis was not performed. Instead, a narrative synthesis was conducted to summarize current evidence on clinical presentation, antibody associations, diagnostic pathways, treatment strategies, and neurological outcomes. The findings were synthesized according to antibody profile, tumor histology, neurological syndrome, treatment, and clinical outcomes.

### 4.3. Quality Assessment

Methodological quality was assessed independently by two reviewers using design-specific JBI critical appraisal checklists. Case reports were evaluated using the JBI Critical Appraisal Checklist for Case Reports, case series using the JBI Critical Appraisal Checklist for Case Series, and observational studies using the corresponding JBI checklist. Each domain was rated as “Yes,” “No,” “Unclear,” or “Not applicable,” and disagreements were resolved through discussion and consensus. Item-level appraisal results are presented in Supplementary Table S2. No study was excluded solely on the basis of methodological quality; the appraisal findings informed interpretation of the evidence.

## 5. Conclusions

The present case highlights that PNSs may constitute the initial manifestation of ovarian malignancy, preceding cancer diagnosis by weeks or even months. The findings demonstrate that ovarian tumor–associated PNSs represent a heterogeneous spectrum of disorders characterized by diverse antibody profiles, clinical phenotypes, immunopathological mechanisms, and outcomes. A particularly unfavorable prognosis was observed in anti-Yo–associated PCD, in which neurological damage is frequently irreversible despite aggressive oncological and immunomodulatory treatment. Owing to nonspecific clinical manifestations, often inconclusive early neuroimaging findings, and the limited availability of comprehensive onconeural antibody testing, these syndromes are likely underrecognized, and their true incidence may therefore be underestimated. Increased clinical awareness, together with early immunological evaluation, may facilitate more timely diagnosis of both the PNS and the underlying ovarian tumor, potentially improving patient management and outcomes.

## Figures and Tables

**Table 1:** Clinical timeline of the diagnostic work-up, oncological treatment, and neurological course in anti-Yo-associated paraneoplastic cerebellar degeneration.

Timepoint	Event Description
**Month 0 (June 2025)**	Progressive dysarthria, gait instability, and impaired coordination. Head CT and CT angiography: no acute cerebrovascular lesion. Retrospective **mRS 2**.
**Month 1 (July 2025)**	Ataxic gait, scanning/explosive speech, four-limb dysmetria, positive Romberg test, brisk reflexes, and left extensor plantar tendency. Brain MRI: no structural cause; **Fazekas 1**, moderate generalized cortical atrophy. CSF: **31 cells/µL**, protein approximately **70 mg/dL**, oligoclonal bands, blood–CSF barrier dysfunction. Anti-Yo1 positive in serum and CSF. **PNS–Care Score 10/10**. Methylprednisolone **1 g i.v./day for 5 days**, without improvement.
**Months 1–2 (July–August 2025)**	**CA-125 37.50 U/mL; HE4 403 pmol/L.** Pelvic ultrasonography: 31 mm left ovarian lesion. Pelvic MRI and CT: a **3.5 × 2.7 cm** para-adnexal mass, Douglas’ pouch and interloop deposits, and ascites. Diagnostic laparoscopy: HGSOC; **CK20−, CK7+, ER+, PAX8+, WT1+, CDX2−; Ki-67 approximately 50%**.
**Month 3 (September 2025)**	**CA-125 45.70 U/mL; HE4 544 pmol/L.** Imaging: progressive intraperitoneal dissemination. Maximal-effort cytoreductive surgery: hysterectomy with bilateral salpingo-oophorectomy, rectal resection, omentectomy, bowel-deposit resection, and pelvic lymph-node excision. Residual macroscopic disease remained.
**Month 4 (October 2025)**	Final diagnosis: HGSOC **pT3 N1a Mx, FIGO IIIC**. Peritoneal/mesenteric deposits up to **3.5 cm**, angioinvasion, and one **5 mm** lymph node metastasis. Postoperative wound dehiscence with evisceration required repeat closure. **CA-125 67.10 U/mL; WHO/ECOG PS 3–4; retrospective mRS 4**.
**Months 5–7 (November 2025–January 2026)**	Four cycles of carboplatin–paclitaxel. Grade 1 neutropenia before cycle 3; subsequent G-CSF prophylaxis. **CA-125 decreased to 20.10 U/mL**. No neurological improvement.
**Month 7 (January 2026)**	Fifth cycle withheld because of **WHO/ECOG PS 3–4, cachexia, muscle wasting, >10% weight loss, grade 2 neutropenia, and persistent neurological disability**. Chemotherapy discontinued; referral to home hospice.
**Month 8 (February 2026)**	Unable to walk or sit independently. Head tremor, multidirectional torsional nystagmus, severe four-limb ataxia, and scanning speech. Gabapentin and baclofen: partial tremor reduction, no functional recovery.
**Month 9 (March 2026)**	Hospitalization for *Clostridioides difficile* infection. Follow-up chest/abdominal/pelvic CT: **no radiological evidence of active malignancy**.
**Month 11 (May 2026)**	**WHO/ECOG PS 4; retrospective mRS 5**. Bedridden, unable to communicate verbally, under home hospice care.
**At manuscript submission**	Patient alive. Biochemical and radiological tumor control without meaningful neurological recovery.

Abbreviations: CA-125, cancer antigen 125; CDX2, caudal-type homeobox 2; CK, cytokeratin; CSF, cerebrospinal fluid; CT, computed tomography; ECOG, Eastern Cooperative Oncology Group; ER, estrogen receptor; FIGO, International Federation of Gynecology and Obstetrics; G-CSF, granulocyte colony-stimulating factor; HE4, human epididymis protein 4; MRI, magnetic resonance imaging; mRS, modified Rankin Scale; PAX8, paired box 8; PNS, paraneoplastic neurological syndrome; PS, performance status; WHO, World Health Organization; WT1, Wilms tumor 1.

**Table 2:** Clinical characteristics, treatments, and outcomes of patients with ovarian tumor-associated PNS involving anti-Yo and other neuronal antibodies.

Author	Study Type	No. of Patients	Ovarian Tumor Type	Antibodies	Main Neurological Manifestations	Oncological Treatment	Immunotherapy	Outcome
**Lehner M.J. et al.** **[6]**	Case series	5	Serous carcinoma	Anti–Yo	Cerebellar ataxia, dysarthria, diplopia	Surgery, chemotherapy	Steroids, IVIG, rituximab	Persistent neurological disability
**Small et al.** **[7]**	Clinicopathological cohort study	26	HGSOC, clear cell carcinoma, sarcomatoid carcinoma	Anti–Yo	Ataxia	Surgery	Not reported	Not reported
**Chatterjee et al.** **[5]**	Prospective cohort study	31	HGSOC	Anti–Yo, Ro52, HARS, 5H6, 4B7, 4H4	Not reported	Surgery, chemotherapy	Not reported	Predicted cancer recurrence
**Butt E. et al.** **[8]**	Case report	1	HGSOC	Negative	Ataxia, dysarthria, nystagmus	Surgery, chemotherapy	None	Cancer remission with severe neurological disability
**Dan Cui et al.** **[9]**	Case report	1	HGSOC	Anti–Yo	Ataxia, dysarthria, nystagmus, dizziness	Surgery, chemotherapy	Steroids, IVIG	Significant clinical improvement
**Stewart et al.** **[10]**	Case report	1	HGSOC	Anti–Ri	Ataxia, opsoclonus–myoclonus, tremor	Surgery, chemotherapy	Steroids	Neurological improvement; late cancer recurrence
**Deac et al.** **[11]**	Case report	1	HGSOC	Anti–Yo	Ataxia, dysarthria, diplopia	Chemotherapy	Steroids, IVIG	Death
**Takashi Shibata et al.** **[12]**	Case report	1	Clear cell carcinoma	Negative	Ataxia, diplopia, dysarthria, dysphagia	Surgery, chemotherapy	IVIG	Complete recovery
**Ju et al.** **[13]**	Case report	1	Adenocarcinoma	Anti–Yo	Ataxia, dysarthria, dysmetria	Surgery	Steroids, IVIG	Clinical improvement
**Huang et al.** **[14]**	Case report and literature review	1	HGSOC	Anti–AMPAR, Anti-Tr/DNER, Anti–recoverin	Encephalopathy, seizures, coma, memory impairment	Surgery, chemotherapy	Steroids, IVIG, PLEX, rituximab	Complete neurological recovery
**M.D. Ray, Rohan Kapoor** **[15]**	Retrospective cohort study	38	HGSOC, endometrioid, clear cell, mucinous, small cell carcinoma	Anti–Yo, Anti–Hu, Anti–Ri, Anti–Ma2, Anti–NMDAR	Ataxia, neuropathy, encephalopathy, opsoclonus–myoclonus	Surgery, chemotherapy	Steroids, IVIG, PLEX	Better prognosis associated with response to PNS treatment
**Giucca A. et al.** **[16]**	Case report and literature review	1	HGSOC	Anti–Yo	Ataxia, nystagmus, diplopia, dizziness	Surgery, chemotherapy	Steroids, PLEX	Death
**Birch J.D. et al.** **[17]**	Case report	1	Carcinosarcoma	Negative	Ataxia, dysarthria, dizziness	Surgery, chemotherapy	None	Progressive neurological deterioration
**Gaughran J. et al.** **[18]**	Case report	1	HGSOC	Anti–Ro52	Trigeminal neuralgia, paresthesia, allodynia	Surgery, chemotherapy	None	Partial neurological recovery
**Juárez-Vignon Whaley et al.** **[19]**	Case report and literature review	1	HGSOC	Anti–CV2	Ataxia, dysarthria, dizziness, tinnitus	Surgery, chemotherapy	Steroids, PLEX	Stable disease
**Farina et al.** **[20]**	Retrospective observational study	1	Clear cell carcinoma	Anti–Yo	Ataxia, dysarthria, nystagmus, paresthesia	Surgery, chemotherapy	Steroids, IVIG, cyclophosphamide, rituximab, natalizumab	Severe neurological disability
**Kumar et al. [21]**	Case report and literature review	1	HGSOC	Anti–Yo	Ataxia, dysarthria, nystagmus, tremor	Chemotherapy	PLEX	Palliative care

Abbreviations: HGSOC—high–grade serous ovarian carcinoma; IVIG—intravenous immunoglobulins; PLEX—plasma exchange; PNS—paraneoplastic neurological syndrome.

**Table 3:** Clinical characteristics, treatments, and outcomes of patients with ovarian tumor–associated anti-NMDAR encephalitis.

Author	Study Type	No. of Patients	Ovarian Tumor Type	Antibodies	Main Neurological Manifestations	Oncological Treatment	Immunotherapy	Outcome
**Kalam et al. [22]**	Case report and literature review	1	Teratoma	Anti–NMDAR	Psychosis, seizures, memory impairment	Surgery	Steroids, IVIG, PLEX	Complete recovery
**Chefdeville et al. [23]**	Research article	3	Mature teratoma; immature teratoma	Anti–NMDAR	Encephalopathy, psychosis, movement disorders	Surgery	Steroids, IVIG, PLEX	Favorable neurological outcome
**Yaguchi et al. [24]**	Single-center observational study	16	Mature teratoma; immature teratoma	Anti–NMDAR	Psychosis, seizures, status epilepticus, catatonia	Surgery	Immunosuppression	Favorable outcome in most patients
**Yi Dai et al. [25]**	Prospective cohort study	1	Mature teratoma; immature teratoma	Anti–NMDAR	Encephalopathy, psychosis, seizures, memory impairment	Surgery	Steroids, IVIG, PLEX	Favorable outcome in most patients
**Lu Zhang et al. [26]**	Cohort study	3	Mature teratoma; immature teratoma	Anti–NMDAR	Encephalopathy, seizures, speech impairment, memory impairment	Surgery	Steroids, IVIG, PLEX, rituximab, cyclophosphamide	Favorable outcome; relapses and deaths reported
**Liang et al. [27]**	Case series and literature review	1	Mature teratoma; immature teratoma	Anti–NMDAR	Psychosis, seizures, cognitive impairment	Surgery, chemotherapy	Steroids, IVIG	Stable condition
**Mutti et al. [28]**	Case report	1	Immature teratoma	Anti–NMDAR	Seizures, psychiatric symptoms, memory impairment	Surgery	Steroids, IVIG, PLEX	Complete recovery
**Eun Hye Cho et al. [29]**	Case report	1	Mucinous cystadenoma	Anti–NMDAR	Encephalopathy	Surgery	Steroids, IVIG	Complete recovery
**Bost et al. [30]**	Retrospective cohort study	6	Mature teratoma; immature teratoma	Anti–NMDAR	Encephalopathy	Surgery, chemotherapy	Steroids, IVIG, PLEX	High mortality
**Chetram D.K. et al. [31]**	Case report	1	Immature teratoma	Anti–NMDAR	Encephalopathy, hallucinations	Surgery, chemotherapy	Steroids	Complete recovery
**Huiyun Jiang et al. [32]**	Retrospective cohort study	3	Mature teratoma; immature teratoma; ND 1	Anti–NMDAR	Seizures, impaired consciousness, motor dysfunction	Surgery	Steroids, IVIG, PLEX, rituximab, cyclophosphamide	Clinical improvement in most patients
**Bai et al. [33]**	Case series	1	Mature teratoma; immature teratoma	Anti–NMDAR	Seizures, dyskinesias, memory impairment	Surgery	Steroids, IVIG, PLEX, rituximab	Significant clinical improvement
**Imataka and Yoshihara [34]**	Case report	1	Immature teratoma	Anti–NMDAR	Memory impairment, seizures, involuntary movements	Surgery	Steroids, IVIG	Complete recovery
**Lee et al. [35]**	Case series	1	Mature teratoma; immature teratoma	Anti–NMDAR	Encephalopathy	Surgery	Steroids, IVIG, PLEX, rituximab	Complete recovery
**Nakamoto et al. [36]**	Case report	1	Immature teratoma	Anti–NMDAR	Confusion, abdominal pain, memory impairment	Surgery	Not reported	Neurological improvement
**Tantipalakorn et al. [37]**	Case report	1	Bilateral mature and immature teratoma	Anti–NMDAR	Encephalopathy, seizures, headache, apnea	Surgery	Steroids, IVIG	Complete recovery

Abbreviations: anti-NMDAR—anti-N-methyl–D–aspartate receptor antibodies; IVIG—intravenous immunoglobulins; PLEX—plasma exchange; ND—no data.

## Data Availability

The data presented in this study are available from the corresponding author upon reasonable request. The data are not publicly available due to privacy and ethical restrictions.

## Supplementary Materials

The following supporting information can be downloaded at: https://www.mdpi.com/article/10.3390/diagnostics16162549/s1, Table S1: Full database-specific search strategies, search parameters, and record retrieval numbers (2016–2026); Table S2: Critical Appraisal of Case Reports and Case Series Using the Joanna Briggs Institute (JBI) Checklists.
