# Supplementary material for: Paraneoplastic Neurological Syndromes Associated with Ovarian Tumors: A Case Report and Systematic Review
Source: Diagnostics (Basel). 2026 Aug 12;16(16):2549. doi: 10.3390/diagnostics16162549 (PMC13512093; doi:10.3390/diagnostics16162549)
Supplement: Supplementary file 1 [file diagnostics-16-02549-s001.zip › diagnostics-4387464-supplementary.pdf]

Table S1. Full database-specific search strategies, search parameters, and record retrieval numbers (2016–2026).

**Database**	**Search Query**	**Date range**	**Records Identified**
**PubMed (MEDLINE)**	("paraneoplastic syndrome*" OR "paraneoplastic neurologic syndrome*" OR "anti-NMDAR encephalitis" OR "cerebellar degeneration" OR "autoimmune disease*" OR "autoimmune response*" OR "tumor-associated antibod*" OR "onconeural antigen*") AND ("gynecologic neoplasm*" OR "ovarian neoplasm*" OR "ovarian carcinoma" OR "ovarian cancer" OR "primary peritoneal carcinoma" OR "peritoneal carcinoma")	Publication date: 2016/01/01 to 2026/03/23;	278

**Scopus**	TITLE-ABS-KEY (("paraneoplastic syndrome*" OR "paraneoplastic neurologic syndrome*" OR "anti-NMDAR encephalitis" OR "cerebellar degeneration" OR "autoimmune disease*" OR "autoimmune response*" OR "tumor-associated antibod*" OR "onconeural antigen*") AND ("gynecologic neoplasm*" OR "ovarian neoplasm*" OR "ovarian carcinoma" OR "ovarian cancer" OR "primary peritoneal carcinoma" OR "peritoneal carcinoma"))	Publication date: 2016 to 2026 (Date of search: March 2026)	232
**Total Identified**			**510**
**Duplicates Removed**			**162**
**Total for Screening**			**348**

Table S2. Critical Appraisal of Case Reports and Case Series Using the Joanna Briggs Institute (JBI) Checklists

Critical Appraisal of Case Reports and Case Series Using the Joanna Briggs Institute (JBI) Checklists

Methodological approach: Case reports were critically appraised using the JBI Critical Appraisal Checklist for Case Reports (8 items), whereas case series were assessed using the JBI Critical Appraisal Checklist for Case Series (10 items). Response options were Yes, No, Unclear, and Not applicable (N/A). In keeping with the domain-based purpose of the JBI checklists, no arbitrary numerical threshold was used to classify overall methodological quality; the overall appraisal was therefore descriptive and based on the pattern of domain-level judgements.

Interpretive note: The appraisal addresses the credibility, transparency, and completeness of reporting within each study design. Even a comprehensively reported case report provides limited support for causal inference because it lacks a comparator group and remains susceptible to selective reporting and publication bias.

Summary of critical appraisal findings

Number	Title	Study design	Yes	No	Unclear	Overall appraisal
1	Utility of paraneoplastic antigens as biomarkers for surveillance and prediction of recurrence in ovarian cancer	Longitudinal diagnostic/prognostic biomarker study	5	1	4	Moderate to high methodological concerns

**Number**	**Title**	**Study design**	**Yes**	**No**	**Unclear**	**Overall appraisal**
2	Paraneoplastic cerebellar degeneration associated with PCA-1 antibodies in established cancer patients	Retrospective case series (n=6)	6	0	3	Moderate methodological concerns
3	A case of acute cerebellar ataxia with two possible etiologies: viral cerebellitis and paraneoplastic antibody-positive ovarian cancer	Case report	7	1	0	Low to moderate methodological concerns
4	Rare case of paraneoplastic cerebellar degeneration secondary to high-grade serous carcinoma of tubo-ovarian origin	Case report	7	1	0	Low to moderate methodological concerns

Number	Title	Study design	Yes	No	Unclear	Overall appraisal
5	Anti-Yo positive and late-onset paraneoplastic cerebellar degeneration associated with ovarian carcinoma	Case report	7	1	0	Low to moderate methodological concerns
6	Paraneoplastic opsoclonus-myoclonus syndrome as a presentation of high grade serous ovarian cancer	Case report	7	1	0	Low to moderate methodological concerns
7	Paraneoplastic cerebellar degeneration associated with anti-Yo antibodies in an ovarian cancer case	Case report	7	0	1	Low to moderate methodological concerns

Number	Title	Study design	Yes	No	Unclear	Overall appraisal
8	Potential effect of intravenous immunoglobulin against paraneoplastic cerebellar degeneration in progressive ovarian cancer	Case report	7	1	0	Low to moderate methodological concerns
9	Paraneoplastic cerebellar ataxia and the paraneoplastic syndromes	Case report	7	1	0	Low to moderate methodological concerns
10	Anti-AMPAR encephalitis developed after ovarian cancer cytoreduction surgery: a case report and literature review	Case report	7	0	1	Low to moderate methodological concerns

Number	Title	Study design	Yes	No	Unclear	Overall appraisal
11	Paraneoplastic syndromes in ovarian carcinoma: A study on clinical spectrum, treatment and survival perspectives	Retrospective prevalence study nested within an ovarian-cancer database	3	6	0	Moderate methodological concerns
12	Anti-Yo paraneoplastic cerebellar degeneration in a patient with stage IV ovarian adenocarcinoma during bevacizumab maintenance therapy	Case report	7	0	1	Low to moderate methodological concerns
13	Paraneoplastic cerebellar degeneration secondary to ovarian carcinosarcoma: a cerebellar conundrum	Case report	7	1	0	Low to moderate methodological concerns

Number	Title	Study design	Yes	No	Unclear	Overall appraisal
14	Paraneoplastic cerebellar degeneration with anti-CV2/CRMP5 antibodies in ovarian cancer	Case report	7	1	0	Low to moderate methodological concerns
15	Trigeminal neuralgia leading to a diagnosis of ovarian cancer	Case report	8	0	0	Low methodological concerns within a case-report design
16	Clinical and tumor features of patients with immune checkpoint inhibitor-related neurological disorders and anti-Yo antibodies	Retrospective case series	8	0	2	Low to moderate methodological concerns
17	Paraneoplastic cerebellar degeneration in occult primary ovarian cancer: a case report and literature review	Case report	8	0	0	Low to moderate methodological concerns

Number	Title	Study design	Yes	No	Unclear	Overall appraisal
18	Anti-NMDAR encephalitis complicating pregnancy	Case report	8	0	0	Low to moderate methodological concerns
19	Immunopathological characterization of ovarian teratomas associated with anti-N-methyl-D-aspartate receptor encephalitis	Retrospective case-control study	4	4	2	Substantial methodological concerns
20	Incidence of anti-NMDAR encephalitis in patients undergoing resection of ovarian teratoma in a single institution	Retrospective prevalence/incidence study	8	0	1	Low to moderate methodological concerns

Number	Title	Study design	Yes	No	Unclear	Overall appraisal
21	Surgical outcomes in patients with anti-N-methyl-D-aspartate receptor encephalitis with ovarian teratoma	Prospective observational cohort study	8	0	0	Low to moderate methodological concerns
22	Anti-N-methyl-D-aspartate receptor encephalitis with accompanying ovarian teratoma in female patients from East China: Clinical features, treatment, and prognostic outcomes	Retrospective cohort study	7	3	1	Moderate methodological concerns

Number	Title	Study design	Yes	No	Unclear	Overall appraisal
23	Teratoma-associated anti-NMDAR encephalitis: two cases report and literature review	Case series	6	1	2	Moderate methodological concerns, principally related to case selection and uncertainty regarding completeness of case inclusion
24	A case of reversible anti-NMDA-receptor encephalitis: neuropsychological and neuroradiological features	Case report	7	1	0	Low to moderate methodological concerns
25	The first case of anti-N-methyl-D-aspartate receptor encephalitis associated with ovarian mucinous cystadenoma: a case report	Case report	7	0	1	Low to moderate methodological concerns

Number	Title	Study design	Yes	No	Unclear	Overall appraisal
26	Malignant tumors in autoimmune encephalitis with anti-NMDA receptor antibodies	Retrospective cohort stud	8	2	1	Moderate methodological concerns
27	Anti-NMDAR encephalitis presenting after immature teratoma resection	Case report	7	0	1	Low to moderate methodological concerns

Number	Title	Study design	Yes	No	Unclear	Overall appraisal
28	Anti-N-Methyl-D-Aspartate Receptor Encephalitis Associated with Ovarian Teratoma in South China: Clinical Features, Treatment, Immunopathology, and Surgical Outcomes of 21 Cases	Case series	8	0	2	Low to moderate methodological concerns: diagnostic and outcome assessment was well standardised, but consecutive recruitment and complete inclusion remained uncertain
29	Treatment principles of ovarian teratoma with anti-N-methyl-D-aspartate receptor encephalitis	Clinical case series	5	1	4	Moderate methodological concerns

**Number**	**Title**	**Study design**	**Yes**	**No**	**Unclear**	**Overall appraisal**
30	Anti-N-methyl-D-aspartate receptor encephalitis associated with ovarian teratoma in Korea: three case reports	Case series	6	1	2	Moderate methodological concerns due to insufficiently reported case-selection methods and uncertainty regarding complete case inclusion
31	Immature ovarian teratoma with anti-NMDA-receptor encephalitis in a 13-year-old Japanese female patient	Case report	8	0	0	Low methodological concerns within a case-report design
32	18F-fluorodeoxyglucose uptake in anti-N-methyl-D-aspartate-receptor encephalitis associated with an immature teratoma	Case report	7	1	0	Low to moderate methodological concerns

Number	Title	Study design	Yes	No	Unclear	Overall appraisal
33	Rapid recovery from catastrophic paraneoplastic anti-NMDAR encephalitis secondary to an ovarian teratoma following ovarian cystectomy	Case report	7	1	0	Low to moderate methodological concerns

Study-level critical appraisals

1. **Utility of Paraneoplastic Antigens as Biomarkers for Surveillance and Prediction of Recurrence in Ovarian Cancer**

Study design: Longitudinal diagnostic/prognostic biomarker study Appraisal tool: JBI Critical Appraisal Checklist for Diagnostic Test Accuracy Studies

No.	JBI appraisal criterion	Judgement	Justification
1	Was a consecutive or random sample of patients enrolled?	Unclear	The report states that patients were entered into a longitudinal ovarian-cancer cohort and that 21 recurrent cases formed the independent test set; however, it does not establish that these participants were selected consecutively or by random sampling.
2	Was a case-control design avoided?	No	The threshold-development stage used a small, deliberately selected set of five recurrent and five non-recurrent patients, while the independent test set contained only recurrent cases. This enriched design

No.	JBI appraisal criterion	Judgement	Justification
			did not provide a representative spectrum for estimating full diagnostic accuracy.
3	Did the study avoid inappropriate exclusions?	Unclear	The eligibility process for the 21-patient test set, reasons for exclusion from the underlying cohort, and availability criteria for serial specimens were not reported in sufficient detail to exclude selection bias.
4	Were the index-test results interpreted without knowledge of the results of the reference standard?	Unclear	Blinding of laboratory personnel to clinical recurrence status, CA125 results, and timing of the specimens was not reported; therefore, review bias cannot be excluded.
5	If a threshold was used, was it pre-specified?	Yes	Antigen-specific thresholds were derived in the training set before application to the independent 21-patient test set, supporting a prespecified analytic rule for validation.
6	Is the reference standard likely to correctly classify the target condition?	Yes	Clinical or radiological evidence of recurrence, supplemented by CA125 and longitudinal medical-record review, constituted a clinically credible reference standard for ovarian-cancer recurrence.
7	Were the reference-standard results interpreted without knowledge of the index-test results?	Yes	The recurrence status was determined from clinical, imaging, and medical-record data collected independently of the experimental Western blot biomarker measurements; no evidence suggests that the index test formed part of the reference standard.
8	Was there an appropriate interval between the index test and reference standard?	Yes	Serial serum samples were explicitly selected from diagnosis, the surveillance period approximately 3–15 months before recurrence, and the time of recurrence. This interval was appropriate for evaluating lead-time prediction.

No.	JBI appraisal criterion	Judgement	Justification
9	Did all patients receive the same reference standard?	Yes	All test-set participants were classified using the same clinical/imaging framework for recurrence and longitudinal disease status.
10	Were all patients included in the analysis?	Unclear	The analysis included the 21 selected recurrent patients, but the report did not provide a complete flow of the source cohort, missing serial samples, or excluded participants. Complete inclusion from the eligible cohort therefore remained uncertain.

Overall methodological appraisal: Moderate to high methodological concerns.

Principal methodological considerations: The longitudinal specimen collection and independent application of prespecified thresholds were strengths. However, the enriched recurrent-case test set, absence of a representative non-recurrent validation group, unclear participant selection, and unreported blinding limit estimates of specificity, predictive value, and generalisability. This publication is not a case report or case series and should not be appraised with either of those JBI checklists.

2. Paraneoplastic Cerebellar Degeneration Associated with PCA-1 Antibodies in Established Cancer Patients

Study design: Retrospective case series (six patients)   Appraisal tool: JBI Critical Appraisal Checklist for Case Series

No.	JBI appraisal criterion	Judgement	Justification
1	Were there clear criteria for inclusion in the case series?	Yes	Eligibility was defined as development of PCA-1-associated paraneoplastic cerebellar degeneration in patients with an established breast or ovarian cancer diagnosis who were not receiving immune-checkpoint immunotherapy,

No.	JBI appraisal criterion	Judgement	Justification
			and cases were identified through institutional medical-record review.
2	Was the condition measured in a standard, reliable way for all participants?	Yes	Across the series, the syndrome was characterised using neurological assessment, neuroimaging, cerebrospinal-fluid evaluation where available, and serum and/or CSF PCA-1 antibody titres. Nevertheless, the extent of testing varied because of the retrospective design.
3	Were valid methods used to identify the condition for all participants?	Yes	PCA-1 antibody testing in serum and/or cerebrospinal fluid, combined with compatible cerebellar manifestations and investigation for alternative neurological causes, provided clinically valid case ascertainment.
4	Did the case series include consecutive participants?	Unclear	The authors did not state whether the six identified patients represented consecutive cases during a defined period; consequently, the potential for selective case inclusion cannot be excluded.
5	Did the case series include all eligible participants?	Unclear	The report did not specify the total number of potentially eligible institutional cases, the search period, or reasons for excluding other patients;

No.	JBI appraisal criterion	Judgement	Justification
			complete inclusion could therefore not be verified.
6	Were participants' demographic characteristics clearly reported?	Yes	Age, sex, primary cancer type, and cancer treatment at neurological presentation were reported for all six patients, including a structured summary table.
7	Was the clinical information of participants clearly reported?	Yes	Neurological symptoms, diagnostic investigations, antibody findings, cancer status, immunosuppressive treatment, and clinical course were described individually for each patient.
8	Were outcomes and follow-up results clearly reported?	Unclear	Follow-up duration and outcomes were reported for most patients, including death, persistent disability, tumour recurrence, cerebellar atrophy, and long-term resolution. However, one patient was lost to follow-up and the reporting was not uniform across all cases.
9	Was the reporting of the presenting site(s) or clinic(s) sufficiently clear?	Yes	The single-institution setting, relevant departments, retrospective chart-review design, and institutional ethics determination were clearly identified.
10	Was the statistical analysis appropriate?	N/A	Formal inferential statistical analysis was not required for this descriptive six-patient case series; the criterion was therefore considered not applicable.

Overall methodological appraisal: Moderate methodological concerns.

Principal methodological considerations: The cases were clinically well characterized and supported by PCA-1 antibody testing. The principal limitations were the absence of an explicit recruitment period, uncertainty regarding consecutive and complete inclusion, heterogeneous diagnostic work-up, and incomplete follow-up for one participant.

3. A case of acute cerebellar ataxia with two possible etiologies: viral cerebellitis and paraneoplastic antibody-positive ovarian cancer (2020)

Study design: Case report Appraisal tool: JBI Critical Appraisal Checklist for Case Reports

No.	JBI appraisal criterion	Judgement	Justification
1	Were the patient's demographic characteristics clearly described?	Yes	The patient's demographic characteristics, including age, sex, relevant medical history, and clinically important baseline information, were clearly reported, allowing appropriate characterisation of the case.
2	Was the patient's history clearly described and presented as a chronological timeline?	Yes	The preceding respiratory illness, acute neurological presentation, diagnostic work-up, treatment sequence, clinical improvement, discharge, and subsequent ovarian surgery were presented in a coherent chronological order.
3	Was the patient's clinical condition at presentation clearly described?	Yes	The acute clinical presentation was described in substantial neurological detail, including ocular findings, dysarthria, limb and gait ataxia, reflexes, motor function, and sensory examination.
4	Were the diagnostic tests or assessment methods and their results clearly described?	Yes	The diagnostic assessment was comprehensively documented and included serial brain MRI,

No.	JBI appraisal criterion	Judgement	Justification
			cerebrospinal-fluid analysis, infectious PCR testing, paraneoplastic antibody testing, serum CA-125, abdominopelvic imaging, and histopathological confirmation of ovarian adenocarcinoma.
5	Was the intervention or treatment procedure clearly described?	Yes	Antiviral therapy, corticosteroid pulse treatment, intravenous immunoglobulin, and subsequent surgical resection of the ovarian mass were clearly described, including treatment duration where reported.
6	Was the patient's post-intervention clinical condition clearly described?	Yes	The marked improvement in cerebellar symptoms, residual subjective dysarthria, follow-up MRI findings, discharge status, and later pathological confirmation after tumour resection were reported.
7	Were adverse events or treatment-related harms identified and described?	No	Adverse events or treatment-related harms were not systematically reported; therefore, this criterion was judged as No according to the JBI appraisal framework.
8	Does the report provide clinically relevant take-away lessons?	Yes	The report provides a clinically relevant lesson that competing infectious and paraneoplastic causes should remain under consideration when evaluating acute cerebellar ataxia.

Overall methodological appraisal: Low to moderate methodological concerns

Principal methodological consideration: Adverse events or treatment-related harms were not systematically reported.

4. Rare case of paraneoplastic cerebellar degeneration secondary to high-grade serous carcinoma of tubo-ovarian origin (2019)

Study design: Case report   Appraisal tool: JBI Critical Appraisal Checklist for Case Reports

No.	JBI appraisal criterion	Judgement	Justification
1	Were the patient’s demographic characteristics clearly described?	Yes	The patient’s demographic characteristics, including age, sex, and clinically relevant baseline information, were clearly described, allowing appropriate characterisation of the reported case.
2	Was the patient’s history clearly described and presented as a chronological timeline?	Yes	The evolution of symptoms, diagnostic work-up, and treatment was reported in a coherent chronological sequence, allowing the clinical course and decision-making process to be followed.
3	Was the patient’s clinical condition at presentation clearly described?	Yes	The clinical presentation was described in sufficient detail, including the principal symptoms, relevant neurological and oncological findings, and functional status at presentation.
4	Were the diagnostic tests or assessment methods and their results clearly described?	Yes	Appropriate diagnostic investigations, including imaging, laboratory analyses, immunological testing, and/or histopathological examination, were reported together with their findings and adequately supported the diagnostic conclusions.
5	Was the intervention or treatment procedure clearly described?	Yes	Antineoplastic, surgical, and/or immunomodulatory management was described in sufficient detail to permit a clear understanding of the therapeutic strategy and its clinical rationale.

No.	JBI appraisal criterion	Judgement	Justification
6	Was the patient’s post-intervention clinical condition clearly described?	Yes	The clinical response to treatment and the subsequent course or follow-up were clearly documented, enabling assessment of post-intervention outcomes.
7	Were adverse events or treatment-related harms identified and described?	No	Adverse events or treatment-related harms were not systematically reported; therefore, this criterion was judged as No according to the JBI appraisal framework.
8	Does the report provide clinically relevant take-away lessons?	Yes	The report provides clinically relevant conclusions that may support recognition of similar presentations and inform diagnostic and therapeutic decision-making.

Overall methodological appraisal: Low to moderate methodological concerns

Principal methodological concern: Adverse events or treatment-related harms were not fully reported.

5. Anti-Yo positive and late-onset paraneoplastic cerebellar degeneration associated with ovarian carcinoma (2017)

Study design: Case report   Appraisal tool: JBI Critical Appraisal Checklist for Case Reports

No.	JBI appraisal criterion	Judgement	Justification
1	Were the patient’s demographic characteristics clearly described?	Yes	The patient’s demographic characteristics, including age, sex, and clinically relevant baseline information, were clearly described, allowing appropriate characterisation of the reported case.

No.	JBI appraisal criterion	Judgement	Justification
2	Was the patient's history clearly described and presented as a chronological timeline?	Yes	The evolution of symptoms, diagnostic work-up, and treatment was reported in a coherent chronological sequence, allowing the clinical course and decision-making process to be followed.
3	Was the patient's clinical condition at presentation clearly described?	Yes	The clinical presentation was described in sufficient detail, including the principal symptoms, relevant neurological and oncological findings, and functional status at presentation.
4	Were the diagnostic tests or assessment methods and their results clearly described?	Yes	Appropriate diagnostic investigations, including imaging, laboratory analyses, immunological testing, and/or histopathological examination, were reported together with their findings and adequately supported the diagnostic conclusions.
5	Was the intervention or treatment procedure clearly described?	Yes	Antineoplastic, surgical, and/or immunomodulatory management was described in sufficient detail to permit a clear understanding of the therapeutic strategy and its clinical rationale.
6	Was the patient's post-intervention clinical condition clearly described?	Yes	The clinical response to treatment and the subsequent course or follow-up were clearly documented, enabling assessment of post-intervention outcomes.

No.	JBI appraisal criterion	Judgement	Justification
7	Were adverse events or treatment-related harms identified and described?	No	Adverse events or treatment-related harms were not systematically reported; therefore, this criterion was judged as No according to the JBI appraisal framework.
8	Does the report provide clinically relevant take-away lessons?	Yes	The report provides clinically relevant conclusions that may support recognition of similar presentations and inform diagnostic and therapeutic decision-making.

Overall methodological appraisal: Low to moderate methodological concerns

Principal methodological concern: Adverse events or treatment-related harms were not fully reported.

6. Paraneoplastic opsoclonus-myoclonus syndrome as a presentation of high grade serous ovarian cancer (2019)

Study design: Case report   Appraisal tool: JBI Critical Appraisal Checklist for Case Reports

No.	JBI appraisal criterion	Judgement	Justification
1	Were the patient's demographic characteristics clearly described?	Yes	The patient's demographic characteristics, including age, sex, and clinically relevant baseline information, were clearly described, allowing appropriate characterisation of the reported case.
2	Was the patient's history clearly described and presented as a chronological timeline?	Yes	The evolution of symptoms, diagnostic work-up, and treatment was reported in a coherent chronological sequence, allowing the clinical course and decision-making process to be followed.

No.	JBI appraisal criterion	Judgement	Justification
3	Was the patient's clinical condition at presentation clearly described?	Yes	The clinical presentation was described in sufficient detail, including the principal symptoms, relevant neurological and oncological findings, and functional status at presentation.
4	Were the diagnostic tests or assessment methods and their results clearly described?	Yes	Appropriate diagnostic investigations, including imaging, laboratory analyses, immunological testing, and/or histopathological examination, were reported together with their findings and adequately supported the diagnostic conclusions.
5	Was the intervention or treatment procedure clearly described?	Yes	Antineoplastic, surgical, and/or immunomodulatory management was described in sufficient detail to permit a clear understanding of the therapeutic strategy and its clinical rationale.
6	Was the patient's post-intervention clinical condition clearly described?	Yes	Treatment response and approximately 10 years of clinical follow-up were reported, providing a particularly comprehensive account of the post-intervention course.
7	Were adverse events or treatment-related harms identified and described?	No	Adverse events or treatment-related harms were not systematically reported; therefore, this criterion was judged as No according to the JBI appraisal framework.

No.	JBI appraisal criterion	Judgement	Justification
8	Does the report provide clinically relevant take-away lessons?	Yes	The report provides clinically relevant conclusions that may support recognition of similar presentations and inform diagnostic and therapeutic decision-making.

Overall methodological appraisal: Low to moderate methodological concerns

Principal methodological concern: Adverse events or treatment-related harms were not fully reported.

7. Paraneoplastic cerebellar degeneration associated with anti-Yo antibodies in an ovarian cancer case (2021)

Study design: Case report   Appraisal tool: JBI Critical Appraisal Checklist for Case Reports

No.	JBI appraisal criterion	Judgement	Justification
1	Were the patient's demographic characteristics clearly described?	Yes	The patient's demographic characteristics, including age, sex, and clinically relevant baseline information, were clearly described, allowing appropriate characterisation of the reported case.
2	Was the patient's history clearly described and presented as a chronological timeline?	Yes	The evolution of symptoms, diagnostic work-up, and treatment was reported in a coherent chronological sequence, allowing the clinical course and decision-making process to be followed.

No.	JBI appraisal criterion	Judgement	Justification
3	Was the patient's clinical condition at presentation clearly described?	Yes	The clinical presentation was described in sufficient detail, including the principal symptoms, relevant neurological and oncological findings, and functional status at presentation.
4	Were the diagnostic tests or assessment methods and their results clearly described?	Yes	Appropriate diagnostic investigations, including imaging, laboratory analyses, immunological testing, and/or histopathological examination, were reported together with their findings and adequately supported the diagnostic conclusions.
5	Was the intervention or treatment procedure clearly described?	Yes	Antineoplastic, surgical, and/or immunomodulatory management was described in sufficient detail to permit a clear understanding of the therapeutic strategy and its clinical rationale.
6	Was the patient's post-intervention clinical condition clearly described?	Yes	The clinical response to treatment and the subsequent course or follow-up were clearly documented, enabling assessment of post-intervention outcomes.
7	Were adverse events or treatment-related harms identified and described?	Unclear	Reporting of adverse events or treatment-related harms was incomplete or insufficiently explicit; consequently, this criterion was judged as Unclear.

No.	JBI appraisal criterion	Judgement	Justification
8	Does the report provide clinically relevant take-away lessons?	Yes	The report provides clinically relevant conclusions that may support recognition of similar presentations and inform diagnostic and therapeutic decision-making.

Overall methodological appraisal: Low to moderate methodological concerns

Principal methodological concern: Adverse events or treatment-related harms were not fully reported.

8. Potential effect of intravenous immunoglobulin against paraneoplastic cerebellar degeneration in progressive ovarian cancer (2020)

Study design: Case report   Appraisal tool: JBI Critical Appraisal Checklist for Case Reports

No.	JBI appraisal criterion	Judgement	Justification
1	Were the patient's demographic characteristics clearly described?	Yes	The patient's demographic characteristics, including age, sex, and clinically relevant baseline information, were clearly described, allowing appropriate characterisation of the reported case.
2	Was the patient's history clearly described and presented as a chronological timeline?	Yes	The evolution of symptoms, diagnostic work-up, and treatment was reported in a coherent chronological sequence, allowing the clinical course and decision-making process to be followed.

No.	JBI appraisal criterion	Judgement	Justification
3	Was the patient's clinical condition at presentation clearly described?	Yes	The clinical presentation was described in sufficient detail, including the principal symptoms, relevant neurological and oncological findings, and functional status at presentation.
4	Were the diagnostic tests or assessment methods and their results clearly described?	Yes	Appropriate diagnostic investigations, including imaging, laboratory analyses, immunological testing, and/or histopathological examination, were reported together with their findings and adequately supported the diagnostic conclusions.
5	Was the intervention or treatment procedure clearly described?	Yes	Antineoplastic, surgical, and/or immunomodulatory management was described in sufficient detail to permit a clear understanding of the therapeutic strategy and its clinical rationale.
6	Was the patient's post-intervention clinical condition clearly described?	Yes	The clinical response to treatment and the subsequent course or follow-up were clearly documented, enabling assessment of post-intervention outcomes.
7	Were adverse events or treatment-related harms identified and described?	No	Adverse events or treatment-related harms were not systematically reported; therefore, this criterion was judged as No according to the JBI appraisal framework.

No.	JBI appraisal criterion	Judgement	Justification
8	Does the report provide clinically relevant take-away lessons?	Yes	The report provides clinically relevant conclusions that may support recognition of similar presentations and inform diagnostic and therapeutic decision-making.

Overall methodological appraisal: Low to moderate methodological concerns

Principal methodological concern: Adverse events or treatment-related harms were not fully reported.

9.Paraneoplastic cerebellar ataxia and the paraneoplastic syndromes (2015)

Study design: Case report   Appraisal tool: JBI Critical Appraisal Checklist for Case Reports

No.	JBI appraisal criterion	Judgement	Justification
1	Were the patient's demographic characteristics clearly described?	Yes	The patient's age, sex, ethnicity, comorbidities, recently identified adnexal mass, and relevant baseline clinical information were clearly reported.
2	Was the patient's history clearly described and presented as a chronological timeline?	Yes	The development of pelvic symptoms, discovery of the adnexal mass, acute neurological deterioration, diagnostic investigations, treatment, and transfer to rehabilitation were presented chronologically.
3	Was the patient's clinical condition at presentation clearly described?	Yes	The presenting neurological condition was described in detail, including headache, diplopia, nystagmus, dysmetria, severe gait

No.	JBI appraisal criterion	Judgement	Justification
			ataxia, reflex findings, and preservation of strength and muscle tone.
4	Were the diagnostic tests or assessment methods and their results clearly described?	Yes	Diagnostic investigations were clearly reported and included tumour biopsy, systemic imaging, serum and cerebrospinal-fluid paraneoplastic antibody testing, cerebrospinal-fluid analysis, serial brain MRI, and exclusion of metastatic disease.
5	Was the intervention or treatment procedure clearly described?	Yes	Carboplatin-paclitaxel chemotherapy, high-dose methylprednisolone, intravenous immunoglobulin, and subsequent rehabilitation and palliative oncological management were adequately described.
6	Was the patient's post-intervention clinical condition clearly described?	Yes	The report documented resolution of headache, slight improvement in diplopia and dysmetria, and transfer to a rehabilitation facility for continued palliative chemotherapy.
7	Were adverse events or treatment-related harms identified and described?	No	Adverse events or treatment-related harms were not systematically identified or described; this criterion was therefore judged as No.
8	Does the report provide clinically relevant take-away lessons?	Yes	The report provides clinically relevant guidance regarding recognition of paraneoplastic neurological syndromes, investigation for an underlying malignancy, and realistic expectations regarding neurological response.

Overall methodological appraisal: Low to moderate methodological concerns

Principal methodological consideration: Adverse events or treatment-related harms were not systematically reported

10. Anti-AMPAR encephalitis developed after ovarian cancer cytoreduction surgery: a case report and literature review (2023)

Study design: Case report   Appraisal tool: JBI Critical Appraisal Checklist for Case Reports

No.	JBI appraisal criterion	Judgement	Justification
1	Were the patient's demographic characteristics clearly described?	Yes	The patient's demographic characteristics, including age, sex, and clinically relevant baseline information, were clearly described, allowing appropriate characterisation of the reported case.
2	Was the patient's history clearly described and presented as a chronological timeline?	Yes	The evolution of symptoms, diagnostic work-up, and treatment was reported in a coherent chronological sequence, allowing the clinical course and decision-making process to be followed.
3	Was the patient's clinical condition at presentation clearly described?	Yes	The clinical presentation was described in sufficient detail, including the principal symptoms, relevant neurological and oncological findings, and functional status at presentation.

No.	JBI appraisal criterion	Judgement	Justification
4	Were the diagnostic tests or assessment methods and their results clearly described?	Yes	Appropriate diagnostic investigations, including imaging, laboratory analyses, immunological testing, and/or histopathological examination, were reported together with their findings and adequately supported the diagnostic conclusions.
5	Was the intervention or treatment procedure clearly described?	Yes	Antineoplastic, surgical, and/or immunomodulatory management was described in sufficient detail to permit a clear understanding of the therapeutic strategy and its clinical rationale.
6	Was the patient's post-intervention clinical condition clearly described?	Yes	The clinical response to treatment and the subsequent course or follow-up were clearly documented, enabling assessment of post-intervention outcomes.
7	Were adverse events or treatment-related harms identified and described?	Unclear	Reporting of adverse events or treatment-related harms was incomplete or insufficiently explicit; consequently, this criterion was judged as Unclear.
8	Does the report provide clinically relevant take-away lessons?	Yes	The report provides clinically relevant conclusions that may support recognition of similar presentations and inform diagnostic and therapeutic decision-making.

Overall methodological appraisal: Low to moderate methodological concerns

Principal methodological concern: Adverse events or treatment-related harms were not fully reported.

11. Paraneoplastic syndromes in ovarian carcinoma: A study on clinical spectrum, treatment and survival perspectives

Study design: Retrospective prevalence study nested within an ovarian-cancer database **Appraisal tool:** JBI Checklist for Prevalence Studies

No.	JBI appraisal criterion	Judgement	Justification
1	Was the sample frame appropriate to address the target population?	**Yes**	The sampling frame comprised a prospectively maintained tertiary-centre database of 376 patients with histologically confirmed ovarian carcinoma treated between 2012 and 2024, which was appropriate for estimating the institutional frequency of paraneoplastic syndromes.
2	Were study participants sampled in an appropriate way?	**Yes**	Eligible patients were identified from the complete ovarian-cancer database using prespecified oncological and paraneoplastic criteria rather than convenience recruitment from isolated reports.
3	Was the sample size adequate?	**Unclear**	The denominator of 376 patients was clinically substantial; however, no a priori precision calculation or justification of the sample size for prevalence estimation was reported.
4	Were the study participants and setting described in sufficient detail?	**Yes**	The setting, study period, eligibility criteria, tumour characteristics, clinical manifestations, and treatment context were described in

No.	JBI appraisal criterion	Judgement	Justification
			sufficient detail to characterise the study population.
5	Was data analysis conducted with sufficient coverage of the identified sample?	**Unclear**	The report states that 38 patients with paraneoplastic syndromes were identified, but it does not fully describe missing data across all six data domains or whether every patient in the sampling frame underwent equivalent syndrome screening.
6	Were valid methods used to identify the condition?	**Unclear**	Ovarian cancer was histologically confirmed, but paraneoplastic syndromes were diagnosed clinically and/or by antibody testing across heterogeneous neurological, endocrine, dermatological, and gastrointestinal presentations. A single uniform case definition was not reported for all syndrome categories.
7	Was the condition measured in a standard and reliable way for all participants?	**Unclear**	The diagnostic work-up included imaging, electrophysiology, cerebrospinal-fluid assessment, and antibody testing as clinically indicated; nevertheless, consistent application of the same ascertainment procedure to all 376 patients was not demonstrated.
8	Was there appropriate statistical analysis?	**Unclear**	Descriptive analyses and Kaplan-Meier methods were reported, but the prevalence estimate was not accompanied by a confidence interval and the handling of potential competing prognostic factors was limited.

No.	JBI appraisal criterion	Judgement	Justification
9	Was the response rate adequate, and if not, was the low response rate managed appropriately?	**Unclear**	The study was database based and did not involve a conventional survey response rate; however, the completeness of records and the extent of missing clinical or follow-up data were not explicitly quantified.

Overall risk-of-bias assessment: Moderate methodological concerns

Principal methodological considerations: The principal concerns relate to heterogeneous case ascertainment for paraneoplastic syndromes, uncertain uniformity of screening across the denominator population, and incomplete reporting of missing data and precision around the prevalence estimate.

12. Anti-Yo paraneoplastic cerebellar degeneration in a patient with stage IV ovarian adenocarcinoma during bevacizumab maintenance therapy (2023)

Study design: Case report   Appraisal tool: JBI Critical Appraisal Checklist for Case Reports

No.	JBI appraisal criterion	Judgement	Justification
1	Were the patient's demographic characteristics clearly described?	Yes	Sex, approximate age (mid-60s), oncological diagnosis, and other clinically relevant baseline characteristics were reported. The absence of an exact age did not preclude adequate characterisation of the case.

No.	JBI appraisal criterion	Judgement	Justification
2	Was the patient’s history clearly described and presented as a chronological timeline?	Yes	The evolution of symptoms, diagnostic work-up, and treatment was reported in a coherent chronological sequence, allowing the clinical course and decision-making process to be followed.
3	Was the patient’s clinical condition at presentation clearly described?	Yes	The clinical presentation was described in sufficient detail, including the principal symptoms, relevant neurological and oncological findings, and functional status at presentation.
4	Were the diagnostic tests or assessment methods and their results clearly described?	Yes	Appropriate diagnostic investigations, including imaging, laboratory analyses, immunological testing, and/or histopathological examination, were reported together with their findings and adequately supported the diagnostic conclusions.
5	Was the intervention or treatment procedure clearly described?	Yes	Antineoplastic, surgical, and/or immunomodulatory management was described in sufficient detail to permit a clear understanding of the therapeutic strategy and its clinical rationale.
6	Was the patient’s post-intervention clinical condition clearly described?	Yes	The clinical response to treatment and the subsequent course or follow-up were clearly documented, enabling assessment of post-intervention outcomes.

No.	JBI appraisal criterion	Judgement	Justification
7	Were adverse events or treatment-related harms identified and described?	Unclear	Reporting of adverse events or treatment-related harms was incomplete or insufficiently explicit; consequently, this criterion was judged as Unclear.
8	Does the report provide clinically relevant take-away lessons?	Yes	The report provides clinically relevant conclusions that may support recognition of similar presentations and inform diagnostic and therapeutic decision-making.

Overall methodological appraisal: Low to moderate methodological concerns

Principal methodological concern: Adverse events or treatment-related harms were not fully reported.

13.Paraneoplastic cerebellar degeneration secondary to ovarian carcinosarcoma: a cerebellar conundrum (2017)

Study design: Case report   Appraisal tool: JBI Critical Appraisal Checklist for Case Reports

No.	JBI appraisal criterion	Judgement	
1	Were the patient's demographic characteristics clearly described?	Yes	The patient's demographic characteristics, including **Justification**age, sex, and clinically relevant baseline information, were clearly described, allowing appropriate characterisation of the reported case.
2	Was the patient's history clearly described and presented as a chronological timeline?	Yes	The evolution of symptoms, diagnostic work-up, and treatment was reported in a coherent chronological sequence, allowing the clinical course and decision-making process to be followed.

No.	JBI appraisal criterion	Judgement	
3	Was the patient's clinical condition at presentation clearly described?	Yes	The clinical presentation was described in sufficient detail, including the principal symptoms, relevant neurological and oncological findings, and functional status at presentation.
4	Were the diagnostic tests or assessment methods and their results clearly described?	Yes	Appropriate diagnostic investigations, including imaging, laboratory analyses, immunological testing, and/or histopathological examination, were reported together with their findings and adequately supported the diagnostic conclusions.
5	Was the intervention or treatment procedure clearly described?	Yes	Antineoplastic, surgical, and/or immunomodulatory management was described in sufficient detail to permit a clear understanding of the therapeutic strategy and its clinical rationale.
6	Was the patient's post-intervention clinical condition clearly described?	Yes	The clinical response to treatment and the subsequent course or follow-up were clearly documented, enabling assessment of post-intervention outcomes.
7	Were adverse events or treatment-related harms identified and described?	No	Adverse events or treatment-related harms were not systematically reported; therefore, this criterion was judged as No according to the JBI appraisal framework.

No.	JBI appraisal criterion	Judgement	
8	Does the report provide clinically relevant take-away lessons?	Yes	The report provides clinically relevant conclusions that may support recognition of similar presentations and inform diagnostic and therapeutic decision-making.

Overall methodological appraisal: Low to moderate methodological concerns

Principal methodological concern: Adverse events or treatment-related harms were not fully reported.

15.Trigeminal Neuralgia Leading to a Diagnosis of Ovarian Cancer: A Timely Coincidence or a Case of Paraneoplastic Syndrome?

Study design: Case report Appraisal tool: JBI Critical Appraisal Checklist for Case Reports

No.	JBI appraisal criterion	Judgement	Justification
1	Were the patient's demographic characteristics clearly described?	Yes	The patient's age, sex, obstetric history, absence of major comorbidity, medication status, and relevant lifestyle characteristics were clearly reported.
2	Was the patient's history clearly described and presented as a chronological timeline?	Yes	The progression from oral discomfort and paraesthesia through dental and neurological assessment, diagnostic imaging, cancer diagnosis, chemotherapy, surgery, and three-month follow-up was presented in a coherent chronological sequence.
3	Was the patient's clinical condition at presentation clearly described?	Yes	The neurological presentation was described in sufficient detail, including the distribution of paraesthesia, allodynia, sensory examination, two-point discrimination, absence of facial

No.	JBI appraisal criterion	Judgement	Justification
			asymmetry, and lack of other cranial-nerve abnormalities.
4	Were the diagnostic tests or assessment methods and their results clearly described?	Yes	The diagnostic evaluation was comprehensive and included oral examination and radiography, neurological examination, laboratory and autoimmune testing, lumbar puncture, contrast-enhanced brain MRI, PET-CT, pelvic MRI, CT staging, CA125 measurement, and histopathological confirmation of high-grade serous carcinoma.
5	Was the intervention or treatment procedure clearly described?	Yes	Neoadjuvant carboplatin-paclitaxel chemotherapy, delayed primary debulking surgery, adjuvant chemotherapy, and symptomatic treatment with gabapentin and amitriptyline were clearly described, including the multidisciplinary decision not to administer high-dose corticosteroids.
6	Was the patient's post-intervention clinical condition clearly described?	Yes	The report documented good postoperative and chemotherapy recovery, persistence of trigeminal-neuralgia symptoms, partial symptomatic response to analgesic therapy, continued daily medication, and planned neurological and rheumatological review.
7	Were adverse events or treatment-related harms identified and described?	Yes	Treatment tolerability and postoperative recovery were explicitly described as favourable, with no major treatment-related complications reported. Persistent neuralgia was clearly distinguished from treatment-related harm.

No.	JBI appraisal criterion	Judgement	Justification
8	Does the report provide clinically relevant take-away lessons?	Yes	The report provides clinically relevant lessons concerning red flags in trigeminal neuralgia, the limitations of remote consultation, the importance of multidisciplinary investigation, and consideration of an occult malignancy or paraneoplastic syndrome.

Overall methodological appraisal: Low methodological concerns within the inherent limitations of a case-report design.

Principal methodological consideration: The report was comprehensive across all JBI domains. Diagnostic attribution remained appropriately cautious because a strongly positive Ro52 antibody left an alternative connective-tissue disorder in the differential diagnosis.

14. Paraneoplastic cerebellar degeneration with anti-CV2/CRMP5 antibodies in ovarian cancer (2021)

Study design: Case report   Appraisal tool: JBI Critical Appraisal Checklist for Case Reports

No.	JBI appraisal criterion	Judgement	Justification
1	Were the patient's demographic characteristics clearly described?	Yes	The patient's demographic characteristics, including age, sex, and clinically relevant baseline information, were clearly described, allowing appropriate characterisation of the reported case.

No.	JBI appraisal criterion	Judgement	Justification
2	Was the patient’s history clearly described and presented as a chronological timeline?	Yes	The evolution of symptoms, diagnostic work-up, and treatment was reported in a coherent chronological sequence, allowing the clinical course and decision-making process to be followed.
3	Was the patient’s clinical condition at presentation clearly described?	Yes	The clinical presentation was described in sufficient detail, including the principal symptoms, relevant neurological and oncological findings, and functional status at presentation.
4	Were the diagnostic tests or assessment methods and their results clearly described?	Yes	Appropriate diagnostic investigations, including imaging, laboratory analyses, immunological testing, and/or histopathological examination, were reported together with their findings and adequately supported the diagnostic conclusions.
5	Was the intervention or treatment procedure clearly described?	Yes	Antineoplastic, surgical, and/or immunomodulatory management was described in sufficient detail to permit a clear understanding of the therapeutic strategy and its clinical rationale.
6	Was the patient’s post-intervention clinical condition clearly described?	Yes	The clinical response to treatment and the subsequent course or follow-up were clearly documented, enabling assessment of post-intervention outcomes.

No.	JBI appraisal criterion	Judgement	Justification
7	Were adverse events or treatment-related harms identified and described?	No	Adverse events or treatment-related harms were not systematically reported; therefore, this criterion was judged as No according to the JBI appraisal framework.
8	Does the report provide clinically relevant take-away lessons?	Yes	The report provides clinically relevant conclusions that may support recognition of similar presentations and inform diagnostic and therapeutic decision-making.

Overall methodological appraisal: Low to moderate methodological concerns

Principal methodological concern: Adverse events or treatment-related harms were not fully reported.

16. Clinical and tumor features of patients with immune checkpoint inhibitor-related neurological disorders and anti-Yo antibodies

Study design: Retrospective case series **Appraisal tool:** JBI Checklist for Case Series

No.	JBI appraisal criterion	Judgement	Justification
1	Were there clear criteria for inclusion in the case series?	**Yes**	The authors defined eligibility as a post-immune checkpoint inhibitor neurological syndrome with anti-Yo antibody positivity confirmed at the national reference centre during 2019–2024.
2	Was the condition measured in a standard and reliable way for all participants?	**Yes**	Anti-Yo status and neurological disability were evaluated using consistent reference-centre laboratory techniques and established clinical scales, including the modified Rankin Scale and CTCAE grading.

No.	JBI appraisal criterion	Judgement	Justification
3	Were valid methods used to identify the condition for all participants?	**Yes**	Anti-Yo antibodies were confirmed using complementary in-house methods, including indirect immunofluorescence, cell-based assays, and immunoblotting for CDR2 and CDR2L.
4	Did the case series include consecutive participants?	**Unclear**	The report states that all eligible patients identified at the reference centre were included, but it does not explicitly describe consecutive enrolment.
5	Did the case series include all eligible participants?	**Yes**	The methods state that all patients meeting the specified phenotype within the reference-centre period were included, supporting complete inclusion within the defined source.
6	Were participants' demographic characteristics clearly reported?	**Yes**	Age, sex, cancer type, immune checkpoint inhibitor exposure, and relevant baseline characteristics were reported for each patient.
7	Was the clinical information of participants clearly reported?	**Yes**	Neurological presentation, temporal relationship to immunotherapy, antibody findings, disability, tumour characteristics, and treatment were described in substantial clinical detail.
8	Were outcomes and follow-up results clearly reported?	**Yes**	Treatment response and neurological outcomes were reported; all cases were severe and the absence of meaningful improvement was clearly documented.

No.	JBI appraisal criterion	Judgement	Justification
9	Was the reporting of the presenting site(s) or clinic(s) sufficiently clear?	**Yes**	The national reference-centre setting, referral context, study period, and participating pathology facilities were adequately described.
10	Was the statistical analysis appropriate?	**N/A**	Given the descriptive nature and sample size of five patients, formal inferential analysis of clinical outcomes was not required; laboratory comparisons were exploratory.

Overall risk-of-bias assessment: Low to moderate methodological concerns

Principal methodological considerations: The case series was well characterised and used robust diagnostic methods. Residual concerns arise primarily from the very small sample, referral-centre selection, and the lack of an explicit statement that enrolment was consecutive.

17. Paraneoplastic cerebellar degeneration in occult primary ovarian cancer: a case report and literature review (2019)

Study design: Case report   Appraisal tool: JBI Critical Appraisal Checklist for Case Reports

No.	JBI appraisal criterion	Judgement	Justification
1	Were the patient’s demographic characteristics clearly described?	Yes	The patient’s demographic characteristics, postmenopausal status, baseline performance status, and relevant clinical background were clearly described.
2	Was the patient’s history clearly described and presented as a chronological timeline?	Yes	The four-month progression of gait and speech disturbance, diagnostic evaluation, antibody testing, identification of metastatic malignancy, treatment, transient response,

No.	JBI appraisal criterion	Judgement	Justification
			complication, and subsequent deterioration were presented as a coherent timeline.
3	Was the patient's clinical condition at presentation clearly described?	Yes	The clinical condition at presentation was described comprehensively, including gait and truncal ataxia, dysarthria, nystagmus, saccadic abnormalities, weakness, reflex abnormalities, tremor, ptosis, autonomic dysfunction, and ECOG performance status.
4	Were the diagnostic tests or assessment methods and their results clearly described?	Yes	The diagnostic work-up was detailed and included cerebrospinal-fluid examination, brain and spinal MRI, neurophysiological and autonomic testing, onconeural antibodies, tumour markers, PET-CT, histopathology, and immunohistochemistry.
5	Was the intervention or treatment procedure clearly described?	Yes	Five cycles of plasmapheresis, paclitaxel-carboplatin chemotherapy, anticoagulation, multidisciplinary review, and transition to supportive care were clearly described.
6	Was the patient's post-intervention clinical condition clearly described?	Yes	Transient neurological improvement after plasmapheresis, subsequent deterioration in general condition and performance status, and the clinical rationale for withholding further cancer-directed treatment were reported.
7	Were adverse events or treatment-related harms identified and described?	Yes	A catheter-associated deep-vein thrombosis was explicitly identified and managed with

No.	JBI appraisal criterion	Judgement	Justification
			rivaroxaban; therefore, treatment-related harm was clearly reported.
8	Does the report provide clinically relevant take-away lessons?	Yes	The report provides clinically relevant lessons regarding the recognition of paraneoplastic cerebellar degeneration as a possible initial manifestation of occult ovarian malignancy and the importance of early diagnostic evaluation.

Overall methodological appraisal: Low to moderate methodological concerns

Principal methodological consideration: Clinically relevant adverse events or treatment-related complications were explicitly reported; no major reporting deficiencies were identified across the remaining JBI domains.

18. Anti-NMDAR encephalitis complicating pregnancy (2018)

Study design: Case report   Appraisal tool: JBI Critical Appraisal Checklist for Case Reports

No.	JBI appraisal criterion	Justification	Justification
1	Were the patient's demographic characteristics clearly described?	Yes	The patient's age, pregnancy status and gestational age, previous health status, and clinically relevant baseline circumstances were clearly documented.
2	Was the patient's history clearly described and presented as a chronological timeline?	Yes	The evolution from behavioural disturbance and psychiatric admission to seizure, respiratory failure, intensive-care management, diagnostic confirmation, surgery, rehabilitation, delivery, and follow-up was presented in a detailed chronological sequence.
3	Was the patient's clinical condition at presentation clearly described?	Yes	The report clearly described the psychiatric, neurological, respiratory, autonomic, and movement-disorder manifestations, including Glasgow Coma Scale score, reflex findings, seizures, dysautonomia, and oromasticatory movements.
4	Were the diagnostic tests or assessment methods and their results clearly described?	Yes	Diagnostic investigations were comprehensively reported and included serial neuroimaging, cerebrospinal-fluid analysis

No.	JBI appraisal criterion	Justification	Justification
			and microbiology, EEG, fetal ultrasonography, pelvic ultrasonography and MRI, serum anti-NMDAR antibody testing, and histopathological confirmation of ovarian teratoma.
5	Was the intervention or treatment procedure clearly described?	Yes	Antimicrobial therapy, antiseizure treatment, mechanical ventilation, corticosteroids, intravenous immunoglobulin, plasma exchange, surgical tumour resection, and rehabilitation were described in sufficient detail.
6	Was the patient's post-intervention clinical condition clearly described?	Yes	Maternal neurological and cognitive recovery, antibody status, rehabilitation course, pregnancy outcome, delivery of a healthy infant, and nine-month maternal and infant follow-up were clearly documented.
7	Were adverse events or treatment-related harms identified and described?	Yes	Clinically important adverse events and complications were explicitly reported, including aspiration pneumonia, respiratory failure, autonomic instability, and cardiac arrest with successful resuscitation.
8	Does the report provide clinically relevant take-away lessons?	Yes	The report provides clinically relevant lessons concerning early recognition and multidisciplinary management of anti-NMDAR encephalitis during pregnancy, including the feasibility of immunotherapy and tumour resection.

Overall methodological appraisal: Low to moderate methodological concerns

Principal methodological consideration: Clinically relevant adverse events or treatment-related complications were explicitly reported; no major reporting deficiencies were identified across the remaining JBI domains.

19. Immunopathological characterization of ovarian teratomas associated with anti-N-methyl-D-aspartate receptor encephalitis

Study design: Retrospective case-control study **Appraisal tool:** JBI Checklist for Case Control Studies

No.	JBI appraisal criterion	Judgement	Justification
1	Were the case and control groups comparable apart from the presence of the condition?	**Unclear**	Both groups had ovarian teratomas, but cases were referred nationally over 2007–2018 whereas controls were all teratomas resected at one regional centre in 2013. Differences in referral pathway, calendar period, tumour maturity, and specimen availability may have affected comparability.
2	Were cases and controls matched appropriately?	**No**	Cases and controls were not individually or frequency matched on age, tumour maturity, calendar period, specimen processing, or other potentially relevant characteristics.
3	Were the same criteria used to identify cases and controls?	**Unclear**	Cases required recognised NMDAR-encephalitis criteria and available tumour tissue. Controls were selected as sporadic teratomas without clinical encephalitis, but

No.	JBI appraisal criterion	Judgement	Justification
			they were not systematically tested for anti-NMDAR antibodies.
4	Was the exposure or characteristic of interest measured in a standard, valid, and reliable way?	**Yes**	Histopathological and immunohistochemical characteristics were assessed using prespecified staining protocols, recognised pathological classifications, and defined semiquantitative criteria.
5	Was the exposure or characteristic measured in the same way for cases and controls?	**Yes**	The same core pathological and immunohistochemical procedures were applied to case and control tumour specimens.
6	Were important confounding factors identified?	**Yes**	The report recognised potentially relevant pathological differences, including tumour maturity, nervous-tissue content, and incomplete tissue embedding.
7	Were strategies to address confounding factors stated?	**No**	No matching, restriction, multivariable adjustment, or other analytical strategy was used to control for differences between the case and control groups.
8	Were outcomes or pathological features assessed in a standard, valid, and reliable way for both groups?	**Unclear**	Experienced pathologists used standard methods and inter-rater agreement was evaluated; however, blinding to case-control status was not reported, creating potential observer bias.
9	Was the exposure period or relevant ascertainment window appropriate?	**Yes**	The ascertainment windows were defined, and controls were followed clinically from 2013

No.	JBI appraisal criterion	Judgement	Justification
			without development of symptoms suggestive of autoimmune encephalitis.
10	Was the statistical analysis appropriate?	**Yes**	Fisher's exact tests, Bonferroni correction for multiple comparisons, and kappa statistics for inter-rater agreement were appropriate for the data structure, although residual confounding remained unaddressed.

Overall risk-of-bias assessment: Moderate to high methodological concerns

Principal methodological considerations: The strongest limitations were non-matched control selection from a different setting and period, incomplete serological exclusion of anti-NMDAR autoimmunity among controls, lack of confounding control, and unreported blinding of pathological assessment.

20. Incidence of anti-NMDAR encephalitis in patients undergoing resection of ovarian teratoma in a single institution

Study design: Retrospective prevalence/incidence study **Appraisal tool:** JBI Checklist for Prevalence Studies

No.	JBI appraisal criterion	Judgement	Justification
1	Was the sample frame appropriate to address the target population?	**Yes**	The pathology registry of a defined single institution over nine years provided an appropriate frame for estimating the frequency of clinically recognised anti-NMDAR encephalitis among patients undergoing teratoma resection.

No.	JBI appraisal criterion	Judgement	Justification
2	Were study participants sampled in an appropriate way?	**Yes**	All patients with a pathological diagnosis of teratoma during the prespecified period were eligible, reducing the likelihood of selective sampling within the institution.
3	Was the sample size adequate?	**Yes**	The denominator included 343 teratoma cases, which was adequate for a descriptive institutional estimate, although the rarity of the outcome limited precision.
4	Were the study participants and setting described in sufficient detail?	**Yes**	The institution, departments, nine-year study period, pathological denominator, and neurological cases were clearly described.
5	Was data analysis conducted with sufficient coverage of the identified sample?	**Yes**	The analysis covered the identified pathology cohort and reported the number of anti-NMDAR encephalitis cases and teratoma-associated cases within the same period.
6	Were valid methods used to identify the condition?	**Yes**	Clinically recognised anti-NMDAR encephalitis was identified using neurological assessment and antibody testing, while teratomas were confirmed pathologically.
7	Was the condition measured in a standard and reliable way for all participants?	**Unclear**	Teratoma pathology was assessed consistently, but the entire denominator population was not systematically screened for neurological symptoms or anti-NMDAR antibodies; clinically mild or unrecognised cases could therefore have been missed.

No.	JBI appraisal criterion	Judgement	Justification
8	Was there appropriate statistical analysis?	**Yes**	The principal calculation was an incidence proportion within the institutional pathology population and was appropriate for the descriptive objective, although confidence intervals were not reported.
9	Was the response rate adequate, and if not, was the low response rate managed appropriately?	**Yes**	The study used routinely collected pathology records rather than participant response, and the denominator appears complete for surgically resected teratomas in the institution.

Overall risk-of-bias assessment: Low to moderate methodological concerns

Principal methodological considerations: The defined pathology denominator and complete institutional sampling were strengths. The main concern is potential under-ascertainment because anti-NMDAR encephalitis was not actively screened across all teratoma patients.

21. Surgical outcomes in patients with anti-N-methyl-D-aspartate receptor encephalitis with ovarian teratoma

Study design: Prospective observational cohort study **Appraisal tool:** JBI Checklist for Cohort Studies

No.	JBI appraisal criterion	Judgement	Justification
1	Were the comparison groups similar and recruited from the same population?	**Unclear**	Patients were prospectively recruited from one centre, but comparisons involving teratoma status and surgery were not based on random allocation and baseline severity differed between groups.
2	Were exposures or group-defining characteristics measured similarly in the groups?	**Yes**	Teratoma status, surgical treatment, timing, and immunotherapy were ascertained using

No.	JBI appraisal criterion	Judgement	Justification
			the same clinical, imaging, operative, and pathological framework.
3	Were exposures or group-defining characteristics measured in a valid and reliable way?	**Yes**	Anti-NMDAR encephalitis was confirmed using validated cell-based antibody testing, and ovarian teratoma was confirmed histopathologically after surgery.
4	Were important confounding factors identified?	**Yes**	The analysis identified clinically relevant prognostic factors, including baseline neurological severity, tumour status, treatment timing, and immunotherapy.
5	Were strategies to address confounding factors stated?	**Yes**	Regression analyses were reported to examine predictors and account for potential confounding, although the limited number of events restricted the robustness of adjustment.
6	Were participants free of the outcome of interest at the start of follow-up, where applicable?	**Yes**	Relapse analyses were initiated after initial clinical improvement or stabilisation, and relapse was explicitly defined as a new or worsening neurological event after at least two months.
7	Were outcomes measured in a valid and reliable way?	**Yes**	Neurological outcome was measured with the modified Rankin Scale using predefined definitions for response, good outcome, and relapse.

No.	JBI appraisal criterion	Judgement	Justification
8	Was the follow-up period reported and sufficiently long for outcomes to occur?	**Yes**	Patients were followed regularly for a mean of approximately 38 months, which was sufficient to observe neurological recovery and relapse.
9	Was follow-up complete, and were reasons for loss to follow-up described?	**Unclear**	Regular follow-up was described, but the report did not provide a complete participant flow with the number and reasons for all losses at each follow-up time point.
10	Were strategies used to address incomplete follow-up?	**Unclear**	No clearly reported sensitivity analysis, weighting procedure, or imputation strategy was used to assess the impact of incomplete follow-up.
11	Was the statistical analysis appropriate?	**Yes**	Kaplan-Meier analysis and regression modelling were broadly appropriate for relapse and prognostic analyses, although estimates were constrained by the small teratoma subgroup and limited event count.

Overall risk-of-bias assessment: Low to moderate methodological concerns

Principal methodological considerations: Prospective recruitment, validated diagnostic methods, predefined outcomes, and extended follow-up were major strengths. Concerns remain regarding non-randomised group differences, incomplete reporting of attrition, and limited statistical power for adjusted analyses.

22. Anti-N-methyl-D-aspartate receptor encephalitis with accompanying ovarian teratoma in female patients from East China: Clinical features, treatment, and prognostic outcomes

Study design: Retrospective cohort study   Appraisal tool: JBI Critical Appraisal Checklist for Cohort Studies

No.	JBI appraisal criterion	Judgement	justification
1	Were the two groups similar and recruited from the same population?	Yes	Patients with and without ovarian teratoma were recruited from the same two tertiary hospitals, during the same period, and under the same diagnostic and eligibility framework for anti-NMDAR encephalitis.
2	Were the exposures measured similarly to assign people to both exposed and unexposed groups?	Yes	All participants underwent systematic tumour screening, and group allocation was based on the presence or absence of a detected ovarian teratoma. Teratomas required postoperative histopathological confirmation.
3	Was the exposure measured in a valid and reliable way?	Yes	Exposure classification was supported by radiological tumour screening and histopathological confirmation after surgery, providing a valid and reliable definition of teratoma status.
4	Were confounding factors identified?	Yes	The report identified clinically relevant group differences and potential prognostic factors, including disease severity at admission, viral prodrome, antibody intensity, treatment intensity, and tumour status.
5	Were strategies to deal with confounding factors stated?	No	No matching, stratified analysis, or multivariable modelling was used to control for baseline disease severity, treatment intensity, age, or other prognostic differences. Residual confounding is therefore likely.

No.	JBI appraisal criterion	Judgement	justification
6	Were the groups free of the outcome at the start of the study or at the moment of exposure?	Yes	The principal longitudinal outcomes, including post-discharge functional status, relapse, full recovery, and mortality, occurred after cohort entry and group classification.
7	Were the outcomes measured in a valid and reliable way?	Unclear	The modified Rankin Scale is a recognised functional outcome measure, and assessments were conducted at predefined intervals by outpatient review or telephone. However, assessor training, standardisation, and blinding to tumour status were not reported.
8	Was the follow-up time reported and sufficient to be long enough for outcomes to occur?	Yes	A minimum follow-up of more than one year was required, except for deaths within the first year, and assessments were scheduled at 3, 6, and 12 months and every six months thereafter. This duration was adequate for recovery and early relapse outcomes.
9	Was follow-up complete, and if not, were the reasons for loss to follow-up described and explored?	No	Seventy-two patients were initially enrolled, whereas 56 who completed at least one year of follow-up were analysed. The approximately 22% attrition was not characterised sufficiently to determine whether losses differed by exposure or prognosis.
10	Were strategies to address incomplete follow-up utilised?	No	No sensitivity analysis, imputation, inverse-probability weighting, or other method was used to assess the effect of incomplete follow-up.
11	Was appropriate statistical analysis used?	Yes	The selected univariable tests were appropriate for the data types and small categorical cells. Nevertheless, the analysis

No.	JBI appraisal criterion	Judgement	justification
			did not adjust for confounding and involved multiple comparisons without a clearly reported multiplicity strategy.

Overall methodological appraisal: Moderate methodological concerns.

Principal methodological considerations: The source population, exposure classification, and duration of follow-up were well defined. The main limitations were substantial attrition, lack of methods to address missing follow-up, absence of adjusted analyses, and uncertainty regarding blinded or standardised outcome assessment.

23. Teratoma-associated anti-NMDAR encephalitis: two cases report and literature review (2017)

Study design: Case series Appraisal tool: JBI Critical Appraisal Checklist for Case Series

No.	JBI appraisal criterion	Judgement	Justification
1	Were clear inclusion criteria specified for the case series?	No	No prespecified inclusion criteria were reported; the article presented two selected cases. The basis on which these cases were chosen was therefore insufficiently transparent.
2	Was the condition measured in a standard and reliable manner for all participants?	Yes	In both patients, the diagnosis was established using consistent clinical criteria, detection of anti-NMDAR antibodies, and confirmation of an ovarian teratoma.

No.	JBI appraisal criterion	Judgement	Justification
3	Were valid methods used to identify the condition in all participants?	Yes	Valid diagnostic methods were used in both cases, including serum and/or cerebrospinal fluid antibody testing, gynaecological imaging, and histopathological assessment.
4	Did the case series include consecutive eligible patients?	Unclear	The authors did not state whether these were consecutive cases managed at the participating centre(s); therefore, the potential for selection bias could not be excluded.
5	Did the case series include all eligible patients during the study period?	Unclear	The report did not provide sufficient information to determine whether all eligible patients during the study period were included; this criterion was therefore judged as Unclear.
6	Were participants' demographic characteristics clearly reported?	Yes	Age and relevant baseline characteristics were reported separately for each of the two patients.
7	Was participants' clinical information clearly reported?	Yes	Symptoms, diagnostic findings, treatment, and the clinical course were described in detail for both patients.
8	Were outcomes and follow-up clearly reported?	Yes	Responses to surgery and immunotherapy and/or chemotherapy, together with clinical status during follow-up, were reported for both patients.

No.	JBI appraisal criterion	Judgement	Justification
9	Were the demographic and clinical characteristics of the study setting(s) clearly described?	Yes	The participating centres or departments and the clinical context were identified, although the characteristics of the underlying referral population were only briefly described.
10	Was the statistical analysis appropriate?	N/A	Given the descriptive nature of this two-patient case series, formal statistical analysis was not required and the criterion was considered not applicable.

Overall methodological appraisal: Moderate methodological concerns, principally related to case selection and uncertainty regarding completeness of case inclusion

Principal methodological concerns: Absence of explicit inclusion criteria and uncertainty regarding consecutive recruitment and complete inclusion of eligible cases.

24.A case of reversible anti-NMDA-receptor encephalitis: neuropsychological and neuroradiological features (2017)

Study design: Case report   Appraisal tool: JBI Critical Appraisal Checklist for Case Reports

No.	JBI appraisal criterion	Judgement	Justification
1	Were the patient’s demographic characteristics clearly described?	Yes	The patient’s age, sex, occupation, substance-use history, reproductive history, family history, and pre-existing pelvic mass were reported in sufficient detail to characterize the case.
2	Was the patient’s history clearly described and presented as a chronological timeline?	Yes	The onset of memory impairment, first seizure, hospital transfer, detailed diagnostic assessment, immunotherapy, surgery, and

No.	JBI appraisal criterion	Judgement	Justification
			serial neuropsychological and neuroradiological follow-up were presented coherently.
3	Was the patient's clinical condition at presentation clearly described?	Yes	The patient's presentation was clearly described, including confusion, impaired orientation, severe short-term episodic memory dysfunction, psychiatric symptoms, seizure activity, and preserved motor examination.
4	Were the diagnostic tests or assessment methods and their results clearly described?	Yes	The diagnostic work-up was particularly detailed and included CT, EEG, 3-T MRI, cerebrospinal-fluid analysis, extensive infectious testing, antibody assessment, tumour evaluation, and standardised serial neuropsychological testing.
5	Was the intervention or treatment procedure clearly described?	Yes	Antiepileptic therapy, high-dose intravenous corticosteroids, surgical treatment of the immature ovarian teratoma, and subsequent medical management were reported with sufficient clarity.
6	Was the patient's post-intervention clinical condition clearly described?	Yes	Serial neuropsychological scores and MRI findings demonstrated rapid and sustained clinical, cognitive, and neuroradiological improvement after treatment, providing a detailed post-intervention assessment.
7	Were adverse events or treatment-related harms identified and described?	No	Adverse events or treatment-related harms were not systematically reported; therefore, this criterion was judged as No.

No.	JBI appraisal criterion	Judgement	Justification
8	Does the report provide clinically relevant take-away lessons?	Yes	The report provides clinically relevant evidence that mild anti-NMDAR encephalitis with prominent amnestic features may be rapidly reversible when recognised and treated promptly.

Overall methodological appraisal: Low to moderate methodological concerns

Principal methodological consideration: Adverse events or treatment-related harms were not systematically reported.

25. The first case of anti-N-methyl-D-aspartate receptor encephalitis associated with ovarian mucinous cystadenoma: a case report (2019)

Study design: Case report   Appraisal tool: JBI Critical Appraisal Checklist for Case Reports

No.	JBI appraisal criterion	Judgement	Justification
1	Were the patient's demographic characteristics clearly described?	Yes	The patient's age, sex, absence of previous medical history, and clinically relevant presenting circumstances were clearly reported.
2	Was the patient's history clearly described and presented as a chronological timeline?	Yes	The progression from influenza-like symptoms and behavioural change to neurological deterioration, diagnostic reconsideration, tumour detection, surgery, immunotherapy, discharge, and rehabilitation follow-up was presented chronologically.
3	Was the patient's clinical condition at presentation clearly described?	Yes	The report clearly described the neuropsychiatric and neurological presentation, including personality change, violent behaviour, confusion, hypoventilation, focal

No.	JBI appraisal criterion	Judgement	Justification
			tonic seizures, reduced muscle tone, impaired ambulation, and need for intubation.
4	Were the diagnostic tests or assessment methods and their results clearly described?	Yes	Diagnostic investigations were clearly reported and included brain CT and MRI, cerebrospinal-fluid analysis and culture, abdominopelvic imaging, transrectal ultrasonography, cell-based anti-NMDAR antibody testing, and histopathological examination.
5	Was the intervention or treatment procedure clearly described?	Yes	Empirical antiviral and corticosteroid therapy, ventilatory and sedative management, laparoscopic right salpingo-oophorectomy, intravenous immunoglobulin, and corticosteroid treatment were described.
6	Was the patient's post-intervention clinical condition clearly described?	Yes	The uncomplicated surgical course, discharge nine days after surgery, and two-month rehabilitation outcome, including recovery of ambulation, communication, and menstrual function, were clearly documented.
7	Were adverse events or treatment-related harms identified and described?	Unclear	The operation was described as uncomplicated, but adverse events and harms associated with the broader intensive-care, immunotherapeutic, and pharmacological management were not systematically assessed; this criterion was therefore judged as Unclear.
8	Does the report provide clinically relevant take-away lessons?	Yes	The report provides a clinically relevant lesson that ovarian neoplasms associated with anti-NMDAR encephalitis may not be limited to teratomas and require histopathological confirmation.

Overall methodological appraisal: Low to moderate methodological concerns

Principal methodological consideration: Reporting of adverse events and treatment-related harms was incomplete or insufficiently systematic.

26.Malignant tumors in autoimmune encephalitis with anti-NMDA receptor antibodies

Study design: Retrospective cohort study **Appraisal tool:** JBI Checklist for Cohort Studies

No.	JBI appraisal criterion	Judgement	PJustification
1	Were the comparison groups similar and recruited from the same population?	**Yes**	Patients were drawn from the same national reference-centre database and met a common laboratory definition of NMDAR-antibody encephalitis.
2	Were exposures or group-defining characteristics measured similarly in the groups?	**Yes**	Tumour status and tumour type were established using the same clinical screening framework and recorded according to a common two-year temporal definition.
3	Were exposures or group-defining characteristics measured in a valid and reliable way?	**Yes**	NMDAR antibodies were confirmed using recognised cerebrospinal-fluid immunohistochemistry and cell-based assays; tumour diagnoses were clinically and pathologically documented.
4	Were important confounding factors identified?	**Yes**	The report described potentially important prognostic factors, including age, sex, tumour

No.	JBI appraisal criterion	Judgement	PJustification
			type, tumour stage, neurological severity, treatment, and timing of tumour diagnosis.
5	Were strategies to address confounding factors stated?	**No**	No multivariable adjustment, matching, or stratified modelling was reported to address substantial differences between patients with benign teratoma, malignant tumours, and no tumour.
6	Were participants free of the outcome of interest at the start of follow-up, where applicable?	**Yes**	Participants were classified at the index encephalitis episode before subsequent recovery or death was assessed.
7	Were outcomes measured in a valid and reliable way?	**Yes**	Neurological outcomes were evaluated with the modified Rankin Scale using a predefined recovery threshold; mortality and tumour progression were also clearly recorded.
8	Was the follow-up period reported and sufficiently long for outcomes to occur?	**Yes**	Follow-up extended over clinically meaningful periods, including several years for many patients, and was adequate to observe neurological recovery and tumour-related mortality.
9	Was follow-up complete, and were reasons for loss to follow-up described?	**No**	Twenty-eight of 280 patients were excluded because clinical data were lacking. The characteristics and outcomes of excluded patients were not reported, creating potential selection bias.

No.	JBI appraisal criterion	Judgement	PJustification
10	Were strategies used to address incomplete follow-up?	**No**	No statistical method was reported to examine the effect of missing records or incomplete follow-up.
11	Was the statistical analysis appropriate?	**Unclear**	Fisher's exact test was appropriate for small categorical comparisons, but the analysis did not control for major prognostic differences and may have produced unstable estimates in very small tumour subgroups.

Overall risk-of-bias assessment: Moderate to high methodological concerns

Principal methodological considerations: Validated diagnosis and clinically meaningful follow-up were strengths. Selection bias from excluded records, very small malignant-tumour subgroups, and absence of confounding adjustment substantially limit comparative inference.

27. Anti-NMDAR encephalitis presenting after immature teratoma resection (2021)

Study design: Case report   Appraisal tool: JBI Critical Appraisal Checklist for Case Reports

No.	JBI appraisal criterion	Judgement	Justification
1	Were the patient's demographic characteristics clearly described?	Yes	The patient's demographic characteristics, including age, sex, and clinically relevant baseline information, were clearly described, allowing appropriate characterisation of the reported case.

No.	JBI appraisal criterion	Judgement	Justification
2	Was the patient's history clearly described and presented as a chronological timeline?	Yes	The evolution of symptoms, diagnostic work-up, and treatment was reported in a coherent chronological sequence, allowing the clinical course and decision-making process to be followed.
3	Was the patient's clinical condition at presentation clearly described?	Yes	The clinical presentation was described in sufficient detail, including the principal symptoms, relevant neurological and oncological findings, and functional status at presentation.
4	Were the diagnostic tests or assessment methods and their results clearly described?	Yes	Appropriate diagnostic investigations, including imaging, laboratory analyses, immunological testing, and/or histopathological examination, were reported together with their findings and adequately supported the diagnostic conclusions.
5	Was the intervention or treatment procedure clearly described?	Yes	Antineoplastic, surgical, and/or immunomodulatory management was described in sufficient detail to permit a clear understanding of the therapeutic strategy and its clinical rationale.
6	Was the patient's post-intervention clinical condition clearly described?	Yes	The clinical response to treatment and the subsequent course or follow-up were clearly documented, enabling assessment of post-intervention outcomes.

No.	JBI appraisal criterion	Judgement	Justification
7	Were adverse events or treatment-related harms identified and described?	Unclear	Reporting of adverse events or treatment-related harms was incomplete or insufficiently explicit; consequently, this criterion was judged as Unclear.
8	Does the report provide clinically relevant take-away lessons?	Yes	The report provides clinically relevant conclusions that may support recognition of similar presentations and inform diagnostic and therapeutic decision-making.

Overall methodological appraisal: Low to moderate methodological concerns

Principal methodological concern: Adverse events or treatment-related harms were not fully reported.

28. Anti-N-Methyl-D-Aspartate Receptor Encephalitis Associated with Ovarian Teratoma in South China: Clinical Features, Treatment, Immunopathology, and Surgical Outcomes of 21 Cases (2021)

Study design: Case series   Appraisal tool: JBI Critical Appraisal Checklist for Case Series

No.	JBI appraisal criterion	Judgement	Justification
1	Were clear inclusion criteria specified for the case series?	Yes	The study population was clearly defined as 21 patients with anti-NMDAR encephalitis and ovarian teratoma who were treated at two centres between 2014 and 2019.
2	Was the condition measured in a standard and reliable manner for all participants?	Yes	The same diagnostic criteria and immunofluorescence-based antibody testing of cerebrospinal fluid and/or serum were applied across participants.

No.	JBI appraisal criterion	Judgement	Justification
3	Were valid methods used to identify the condition in all participants?	Yes	The diagnostic, imaging, and histopathological methods, together with modified Rankin Scale assessment, were appropriately described and were suitable for identifying and characterising the condition.
4	Did the case series include consecutive eligible patients?	Unclear	The article did not explicitly state that consecutive eligible patients were enrolled; accordingly, the possibility of selection bias could not be excluded.
5	Did the case series include all eligible patients during the study period?	Unclear	No participant flow diagram or denominator for all eligible patients was provided; consequently, complete case inclusion could not be confirmed.
6	Were participants' demographic characteristics clearly reported?	Yes	Age, symptom distribution, and relevant clinical characteristics were reported for the full cohort.
7	Was participants' clinical information clearly reported?	Yes	Symptoms, ancillary investigations, treatment, pathological findings, and disease severity were reported in substantial detail.
8	Were outcomes and follow-up clearly reported?	Yes	Outcomes and follow-up time points were defined, and postoperative results, clinical improvement, and relapse were reported.
9	Were the demographic and clinical characteristics of the study setting(s) clearly described?	Yes	The two tertiary hospitals, geographical region, and recruitment period were clearly reported.

No.	JBI appraisal criterion	Judgement	Justification
10	Was the statistical analysis appropriate?	Yes	The comparative analyses were appropriate to the study objectives. Nevertheless, the small sample size limits statistical precision and increases the likelihood of unstable estimates.

Overall methodological appraisal: Low to moderate methodological concerns -good standardization, but uncertain consecutiveness and completeness of inclusion

Principal methodological concerns: Consecutive recruitment and complete inclusion of eligible cases were not clearly established.

29.Treatment principles of ovarian teratoma with anti-N-methyl-D-aspartate receptor encephalitis

Study design: Clinical case series **Appraisal tool:** JBI Checklist for Case Series

No.	JBI appraisal criterion	Judgement	Justification
1	Were there clear criteria for inclusion in the case series?	**Unclear**	The report stated that ten patients with ovarian teratoma-associated anti-NMDAR encephalitis were enrolled between June 2011 and October 2014, but formal inclusion and exclusion criteria were not fully specified.
2	Was the condition measured in a standard and reliable way for all participants?	**Yes**	All patients were described as having ovarian teratoma associated with anti-NMDAR encephalitis and underwent a broadly consistent surgical and immunotherapeutic management pathway.

No.	JBI appraisal criterion	Judgement	Justification
3	Were valid methods used to identify the condition for all participants?	**Unclear**	The report did not provide a sufficiently detailed, uniform diagnostic algorithm for anti-NMDAR encephalitis across all participants, although the clinical diagnosis and tumour association were described.
4	Did the case series include consecutive participants?	**Unclear**	The authors did not state whether the ten patients represented consecutive eligible cases.
5	Did the case series include all eligible participants?	**Unclear**	It was not reported whether all eligible patients treated at the institution during the study period were included.
6	Were participants' demographic characteristics clearly reported?	**Yes**	Age, sex, tumour laterality, pathological maturity, and selected clinical characteristics were reported for the patients.
7	Was the clinical information of participants clearly reported?	**Yes**	Neurological and psychiatric manifestations, surgical care, immunotherapy, perioperative management, and selected complications were described in considerable detail.
8	Were outcomes and follow-up results clearly reported?	**Yes**	Postoperative symptom improvement, hospital status, recurrence, and follow-up outcomes were reported, although follow-up duration varied and one patient remained hospitalised at manuscript preparation.
9	Was the reporting of the presenting site(s) or clinic(s) sufficiently clear?	**Yes**	The hospital, study period, surgical context, and treatment setting were identified clearly.

No.	JBI appraisal criterion	Judgement	Justification
10	Was the statistical analysis appropriate?	**N/A**	The study was primarily a descriptive series of ten patients; formal comparative statistical analysis was not essential to its stated descriptive purpose.

Overall risk-of-bias assessment: Moderate methodological concerns

Principal methodological considerations: Clinical management and outcomes were reported in detail, but uncertainty regarding eligibility criteria, consecutive recruitment, complete inclusion, and uniform diagnostic ascertainment limits confidence in representativeness.

30. Anti-N-methyl-D-aspartate receptor encephalitis associated with ovarian teratoma in Korea: three case reports (2021)

Study design: Case series   Appraisal tool: JBI Critical Appraisal Checklist for Case Series

No.	JBI appraisal criterion	Judgement	Justification
1	Were clear inclusion criteria specified for the case series?	No	Formal inclusion criteria were not reported; the article presented three cases without specifying the framework used to select them.
2	Was the condition measured in a standard and reliable manner for all participants?	Yes	A consistent diagnostic approach to anti-NMDAR encephalitis and ovarian tumour assessment was applied across all three patients.
3	Were valid methods used to identify the condition in all participants?	Yes	The diagnosis was supported by antibody testing, imaging, and histopathological examination following tumour resection.

No.	JBI appraisal criterion	Judgement	Justification
4	Did the case series include consecutive eligible patients?	Unclear	The authors did not explicitly report whether the patients represented consecutive eligible cases; consequently, this criterion was judged as Unclear.
5	Did the case series include all eligible patients during the study period?	Unclear	The report did not establish that all eligible cases arising during the relevant period were included, leaving the completeness of case ascertainment uncertain.
6	Were participants' demographic characteristics clearly reported?	Yes	Demographic characteristics were reported separately for each of the three patients.
7	Was participants' clinical information clearly reported?	Yes	The clinical course, diagnostic assessment, and treatment were described for each patient.
8	Were outcomes and follow-up clearly reported?	Yes	Post-treatment improvement and subsequent follow-up were reported, although the duration of follow-up varied across cases.
9	Were the demographic and clinical characteristics of the study setting(s) clearly described?	Yes	The hospital setting and the clinical context of the Korean case series were clearly identified.
10	Was the statistical analysis appropriate?	N/A	Formal statistical analysis was not required for this descriptive three-patient case series; the criterion was therefore considered not applicable.

Overall methodological appraisal: Moderate methodological concerns due to insufficiently reported case-selection methods and uncertainty regarding complete case inclusion

Principal methodological concerns: Absence of explicit inclusion criteria and uncertainty regarding consecutive recruitment and complete inclusion of eligible cases.

31. Immature Ovarian Teratoma with Anti-NMDA-Receptor Encephalitis in a 13-Year-Old Japanese Female Patient

Study design: Case report   Appraisal tool: JBI Critical Appraisal Checklist for Case Reports

No.	JBI appraisal criterion	Judgement	Justification
1	Were the patient's demographic characteristics clearly described?	Yes	The patient's age, sex, nationality, preceding respiratory illness, and clinically relevant baseline context were clearly reported.
2	Was the patient's history clearly described and presented as a chronological timeline?	Yes	The report presented a coherent sequence from the preceding common cold and psychiatric symptoms through neurological deterioration, diagnostic testing, tumour detection, ovariectomy, immunotherapy, hospital discharge, and two-year follow-up.
3	Was the patient's clinical condition at presentation clearly described?	Yes	The initial psychiatric and neurological condition was clearly described, including memory impairment, insomnia, binge eating, abnormal mouth and hand movements, constipation, declining consciousness, seizures, daytime apathy, nocturnal apnoea, and substantial weight loss.
4	Were the diagnostic tests or assessment methods and their results clearly described?	Yes	Diagnostic investigations and findings were reported in sufficient detail, including blood and autoimmune testing, tumour markers, EEG,

No.	JBI appraisal criterion	Judgement	Justification
			cerebrospinal-fluid analysis, brain CT and MRI, abdominal ultrasonography and MRI, serum and CSF anti-NMDAR antibodies, and histopathological grading of the teratoma.
5	Was the intervention or treatment procedure clearly described?	Yes	Empirical acyclovir and cefotaxime, urgent right ovariectomy, repeated methylprednisolone pulse therapy, high-dose intravenous immunoglobulin, and valproic acid were described with dose and duration where available.
6	Was the patient's post-intervention clinical condition clearly described?	Yes	The subsequent improvement in mental state and memory, discharge after two months, pathological confirmation, normal-range IQ at two years, discontinuation of antiepileptic medication, and absence of neurological sequelae were clearly reported.
7	Were adverse events or treatment-related harms identified and described?	Yes	Clinically important complications and adverse clinical events were explicitly reported, including nocturnal apnoea, loss of more than 10% of body weight, and repeated generalised seizures requiring valproic acid.
8	Does the report provide clinically relevant take-away lessons?	Yes	The report provides a clinically relevant lesson that sudden psychiatric symptoms in a young female patient should prompt evaluation for autoimmune encephalitis and an associated ovarian teratoma, with early tumour removal and immunotherapy.

Overall methodological appraisal: Low methodological concerns within the inherent limitations of a case-report design.

Principal methodological consideration: Despite its concise format, the report adequately covered all JBI domains, including treatment-related complications and extended neurological follow-up.

32. 18F-fluorodeoxyglucose uptake in anti-N-methyl-D-aspartate-receptor encephalitis associated with an immature teratoma (2017)

Study design: Case report   Appraisal tool: JBI Critical Appraisal Checklist for Case Reports

No.	JBI appraisal criterion	Judgement	Justification
1	Were the patient’s demographic characteristics clearly described?	Yes	The patient’s age, sex, and principal baseline clinical circumstances were clearly reported, allowing adequate characterisation of the case.
2	Was the patient’s history clearly described and presented as a chronological timeline?	Yes	The sequence from acute abdominal pain and identification of an ovarian mass to PET imaging, subsequent cognitive symptoms, tumour resection, antibody confirmation, and neurological improvement was presented chronologically.
3	Was the patient’s clinical condition at presentation clearly described?	Yes	The post-imaging neurological presentation, including disorientation and short-term memory impairment, was clearly described, although the report was intentionally concise.
4	Were the diagnostic tests or assessment methods and their results clearly described?	Yes	The report clearly described pelvic ultrasonography, MRI, whole-body FDG PET/CT, dedicated review of cerebral PET findings, cerebrospinal-fluid anti-NMDAR antibody testing, and histopathological diagnosis of an immature teratoma.
5	Was the intervention or treatment procedure clearly described?	Yes	Surgical resection of the ovarian tumour and the relevant diagnostic and therapeutic

No.	JBI appraisal criterion	Judgement	Justification
			sequence were adequately reported for the purpose of this imaging-focused case report.
6	Was the patient's post-intervention clinical condition clearly described?	Yes	Neurological improvement following surgery was explicitly documented, allowing assessment of the post-intervention course.
7	Were adverse events or treatment-related harms identified and described?	No	Adverse events or treatment-related harms were not reported; therefore, this criterion was judged as No.
8	Does the report provide clinically relevant take-away lessons?	Yes	The report provides a clinically relevant imaging lesson by demonstrating unexpected focal cerebral FDG uptake in anti-NMDAR encephalitis associated with an immature ovarian teratoma.

Overall methodological appraisal: Low to moderate methodological concerns

Principal methodological consideration: Adverse events or treatment-related harms were not systematically reported

33. Rapid recovery from catastrophic paraneoplastic anti-NMDAR encephalitis secondary to an ovarian teratoma following ovarian cystectomy (2016)

Study design: Case report   Appraisal tool: JBI Critical Appraisal Checklist for Case Reports

**No.**	**JBI appraisal criterion**	**Judgement**	**Justification**
1	Were the patient's demographic characteristics clearly described?	Yes	The patient's demographic characteristics, including age, sex, and clinically relevant baseline information, were clearly described, allowing appropriate characterisation of the reported case.
2	Was the patient's history clearly described and presented as a chronological timeline?	Yes	The evolution of symptoms, diagnostic work-up, and treatment was reported in a coherent chronological sequence, allowing the clinical course and decision-making process to be followed.
3	Was the patient's clinical condition at presentation clearly described?	Yes	The clinical presentation was described in sufficient detail, including the principal symptoms, relevant neurological and oncological findings, and functional status at presentation.
4	Were the diagnostic tests or assessment methods and their results clearly described?	Yes	Appropriate diagnostic investigations, including imaging, laboratory analyses, immunological testing, and/or histopathological examination, were reported together with their findings and adequately supported the diagnostic conclusions.

No.	JBI appraisal criterion	Judgement	Justification
5	Was the intervention or treatment procedure clearly described?	Yes	Antineoplastic, surgical, and/or immunomodulatory management was described in sufficient detail to permit a clear understanding of the therapeutic strategy and its clinical rationale.
6	Was the patient's post-intervention clinical condition clearly described?	Yes	The clinical response to treatment and the subsequent course or follow-up were clearly documented, enabling assessment of post-intervention outcomes.
7	Were adverse events or treatment-related harms identified and described?	No	Adverse events or treatment-related harms were not systematically reported; therefore, this criterion was judged as No according to the JBI appraisal framework.
8	Does the report provide clinically relevant take-away lessons?	Yes	The report provides clinically relevant conclusions that may support recognition of similar presentations and inform diagnostic and therapeutic decision-making.

Overall methodological appraisal: Low to moderate methodological concerns

Principal methodological concern: Adverse events or treatment-related harms were not fully reported.

Across the individual case reports, the most frequent limitation was incomplete or absent reporting of adverse events and treatment-related harms. Other JBI domains were generally well addressed, particularly the chronological description of the clinical course, immunological and imaging-based diagnostic assessment, and reporting of treatment response.

In the small case series comprising two or three patients, the principal methodological concerns arose from the absence of explicit inclusion criteria and insufficient information on consecutive recruitment and complete inclusion of eligible cases.

The retrospective 21-patient case series used more standardised diagnostic criteria and outcome measures; however, consecutive recruitment and complete case ascertainment were not unequivocally demonstrated.
